# Effects of Mediterranean diet or Mindfulness-Based Stress Reduction during pregnancy on maternal gut and vaginal microbiota: a subanalysis of the Improving Mothers for a better PrenAtal Care Trial BarCeloNa (IMPACT BCN) trial

**DOI:** 10.1016/j.ajcnut.2025.07.030

**Published:** 2025-08-05

**Authors:** Marta Selma-Royo, Fàtima Crispi, Sara Castro-Barquero, Irene Casas, Marta Larroya, Mariona Genero, Cristina Paules, Leticia Benitez, Lina Youssef, Rosalia Pascal, Noelia Encabo, Ayako Nakaki, Andrés Martín-Asuero, M Teresa Oller-Guzmán, Angela Arranz, Eduard Vieta, Rosa Casas, Ramon Estruch, Eduard Gratacos, Maria Carmen Collado, Francesca Crovetto

**Affiliations:** 1 BCNatal (Hospital Clínic and Hospital Sant Joan de Déu), University of Barcelona, Barcelona, Spain; 2 Institut d'Investigacions Biomèdiques August Pi i Sunyer (IDIBAPS), Barcelona, Spain; 3 Centre for Biomedical Research on Rare Diseases (CIBER-ER), Madrid, Spain; 4 Department of Nutrition, Harvard TH Chan School of Public Health, Boston, MA, United States; 5 Institut de Recerca Sant Joan de Déu (IRSJD), Barcelona, Spain; 6 Instituto de Investigación Sanitaria Aragón (IISAragon), Red de Salud Materno Infantil y del Desarrollo (SAMID), RETICS, Instituto de Salud Carlos III (ISCIII), Subdirección General de Evaluación y Fomento de la Investigación y Fondo Europeo de Desarrollo Regional (FEDER), Zaragoza, Spain; 7 Josep Carreras Leukaemia Research Institute, Hospital Clinic/University of Barcelona Campus, Barcelona, Spain; 8 Primary care interventions to prevent maternal and child chronic diseases of perinatal and developmental origin RD21/0012/0003, Instituto de Salud Carlos III, Spain; 9 Instituto esMindfulness, Barcelona, Spain; 10 Department of Psychiatry and Psychology, Hospital Clinic, Neuroscience Institute, IDIBAPS, University of Barcelona, CIBERSAM, Barcelona, Spain; 11 Centro de Investigación Biomédica en Red de Fisiopatología de la Obesidad y Nutrición (CIBEROBN), Instituto de Salud Carlos III, Madrid, Spain; 12 Institut de Recerca en Nutrició i Seguretat Alimentaria (INSA-UB). University of Barcelona, Barcelona, Spain; 13 Department of Internal Medicine Hospital Clinic, University of Barcelona, Barcelona, Spain; 14 Institute of Agrochemistry and Food Technology-National Research Council (IATA-CSIC), Paterna, Valencia, Spain; 15 Facultat de Medicina i Ciències de la Salut, University of Barcelona, Spain

**Keywords:** microbiota, pregnancy, Mediterranean diet, Stress reduction, randomized clinical trial

## Abstract

**Background:**

Despite the importance of gestation period for human health, studies addressing the impact of maternal microbiota on its progression and its modulation by maternal lifestyle are scarce. Although most of the evidence in the field comes from observational studies, we recently described how some lifestyle interventions during pregnancy reduced the small-for-gestational-age (SGA) incidence. We hypothesized the pregnant individual’s microbiome modulation as potential mechanism by which lifestyle interventions could impact gestation progression.

**Objectives:**

The objectives of this study was to investigate the effect of pregnancy lifestyle interventions, based on a Mediterranean dietary pattern (MD) or Stress Reduction program (SR) that proved to be beneficial in reducing the SGA incidence, on the pregnant individuals’ gut and vaginal microbiota as exploratory outcomes.

**Methods:**

In a random subsample (*n* = 351) from a trial including pregnancies randomly allocated into an MD intervention, an SR program, or non-intervention, maternal fecal/vaginal samples were collected at the end of the interventions, and in a subset (*n* = 85) also at recruitment. Microbiota was profiled by 16S rRNA gene sequencing. Multivariate models evaluated the associations of microbiota with the interventions.

**Results:**

Mothers included in the study presented a similar gut microbiota profile before the intervention; pregnancy MD intervention influenced the overall structure of gut microbiota, (R2 = 0.008, F = 1.885, *P* = 0.002), which resulted in enrichment in Firmicutes phylum (Coeff = 0.06, 95% confidence interval [CI]: 0.002, 0.118), related to the increment in health-associated taxa (Lachnospiraceae/Ruminococcaceae families) and other SCFA producers; and diminishment in *Campylobacter* genus (Coeff = −0.91, 95% CI: −1.361, −0.459). The link of the SR program with gut microbiota was more limited; however, some key components from these families were also affected. Microbial diversity of gut microbiota decreased as pregnancy progressed, with this effect more observable in the mothers that followed the interventions. The interventions have a negligible association with the vaginal microbiota.

**Conclusions:**

Pregnancy lifestyle interventions influence maternal gut microbiota.

The trial was registered at clinicaltrials.gov Identifier as NCT03166332.

**Clinical Trial Information:**

Date of Institutional Review Board approval and registration: 16 December 2016; 19 April 2017; Date of initial participant enrollment and date of first outcome (i.e., delivery of the pregnant individual enrolled): 1 February 2017; 10 May 2017.

## Introduction

The importance of gut microbiota for human health has become more evident since specific links between alterations of microbiota composition and several pathological conditions have been described [1]. During pregnancy, many physiological changes such as metabolism, hormonal cascades, immunologic events, etc., occur to promote the development of the fetus and prepare the mother for childbirth and lactation. Recent studies have reported changes in vaginal [2] and gut [3] microbiota during pregnancy. Maternal microbiota represents the most important microbial source for the neonatal microbiota colonization process and thus, could also be involved in neonatal development with implications on future adult health [4], including the risk of obesity, metabolic syndrome, diabetes, and allergy-related problems [5]. Although gestational and perinatal factors that shape the maternal microbiota are yet to be truly defined, environmental conditions such as diet and lifestyle seem relevant in adult microbiota composition [6]. It is reasonable to hypothesize that changes in nutrient intake directly affecting gut bacteria metabolism and/or stress-related signals released by the host and reaching the intestine, may be associated with modulations of the female gut microbiome.

Although few studies reported influences of female lifestyle during pregnancy on microbiota composition, all of them came from observational studies with no application of structured interventions [7,8]. Pregnancy is a unique period in which healthcare interventions to improve the mother’s well-being can affect both maternal and fetal health. Thus, strategies during pregnancy may provide a pivotal opportunity to positively affect the outcome of the pregnancy itself, together with the mother and offspring’s health [9].

In this context, we aimed to assess the effect of structured lifestyle interventions during pregnancy, based on Mediterranean diet or Mindfulness-Based Stress Reduction, on pregnant individuals’ microbiota, both gut and vaginal niches.

## Methods

### Study design, population, and ethics

Pregnant individuals were randomly selected from the **I**mproving **M**others for a better **P**ren**A**tal **C**are **T**rial **B**ar**C**elo**N**a (IMPACT BCN), which was a parallel, randomized clinical trial conducted at BCNatal (Hospital Clínic and Hospital Sant Joan de Déu), a large referral center for maternal-fetal and neonatal medicine in Barcelona, Spain. Details of the trial are provided in the protocol [10], approved by the Institutional Review Board (HCB-2016-0830) before any participant enrollment, and registered (ClinicalTrials.gov identifier NCT03166332) before any outcome occurred. All individuals who agreed to participate provided written informed consent before randomization.

Participants were screened for eligibility during routine second-trimester ultrasound scans (19–23.6 wk of gestation) under the criteria of the Royal College of Obstetrics and Gynecologists for being at high risk of developing a small-for-gestational-age (SGA) newborn, reported in Supplemental materials [11]. Participants who agreed to join the trial were randomly assigned 1:1:1 to 1 of the 3 study groups: a Mediterranean diet (Med Diet) supplemented with extra-virgin olive oil and walnuts; a Stress reduction intervention based on mindfulness (Stress reduction); or usual care without any intervention (Usual Care group – non intervention). The primary outcome of the trial was the prevalence of SGA newborns, and results can be found elsewhere [9]. Inclusion criteria were age ≥18 y, fluency in Spanish language, singleton pregnancy, positive fetal heart rate at the time of ultrasonography, and high risk of newborns with SGA according to the adapted criteria of the Royal College of Obstetricians and Gynaecologists for SGA [11] (see Supplemental material). Exclusion criteria were fetal anomalies, including chromosomal abnormalities, structural malformations, and congenital infections, detected prenatally; neonatal malformations or congenital anomalies diagnosed after birth; inability to perform additional visits; participation in another trial; and maternal intellectual disability or other mental or major psychiatric disorders requiring therapy during pregnancy.

The impact of these interventions on gut/vaginal microbiota composition and diversity was an exploratory outcome evaluated according to the study design [10]. Recruitment started on 1 February 2017 and ended on 10 October 2019, with a follow-up until delivery up to 1 March 2020 and a subsequent follow-up for secondary outcomes until 31 May 2022.

### Sample size

Among participants recruited for the IMPACT BCN trial, ≤20% of each study group were randomly selected to evaluate the maternal gut and vaginal microbiota at the end of the intervention (34–36 wk’ gestation), assuming a type I error of 5% and aiming for a power of 80% for finding any differences on the gut microbiota among involved participants. Thus, among the 1221 randomly assigned pregnancies, 351 pregnant individuals were randomly selected among study groups (Usual Care *n* = 120, Med Diet *n* = 109, Stress reduction *n* = 122) for the study of maternal gut and vaginal microbiota. Additionally, in a midstudy decision aimed at improving the analysis, maternal gut and vaginal microbiota were also collected at enrollment from 85 females, before any intervention (Usual Care *n* = 26, Med Diet *n* = 24, Stress reduction *n* = 35).

### Interventions and measurements

The interventions were non-pharmacologic, based on counseling and behavioral training.

#### Mediterranean diet program

The intervention aimed to change the general dietary pattern, instead of focusing on changes in single foods or macronutrients. All participants received extra-virgin olive oil (2 L every month) and 15 g of walnuts daily (450 g every month) at no cost. Dietitians conducted face-to-face interviews at enrollment, and monthly until the end of the intervention (34–36 wks’ gestation). Additional details of the intervention are provided elsewhere [10].

#### Mindfulness-based stress reduction program

The Stress reduction program was based on the program described by Kabat-Zinn and later adopted by health institutions. It was consistent with previously described Mindfulness-Based Stress Reduction programs for adults: an 8-wk program of weekly 2.5-h sessions, one full-day session, and daily home practice, with specific adaptation to pregnancy. The sessions included didactic presentations, formal 45-min meditation practices with various mindfulness techniques, mindful yoga, body awareness, and group discussion. Additional details of the intervention are provided elsewhere [10].

#### Usual care (nonintervention group)

Pregnant individuals randomly assigned to this group received usual pregnancy care as per institutional protocols.

### Data collection

**FIGURE 1:**
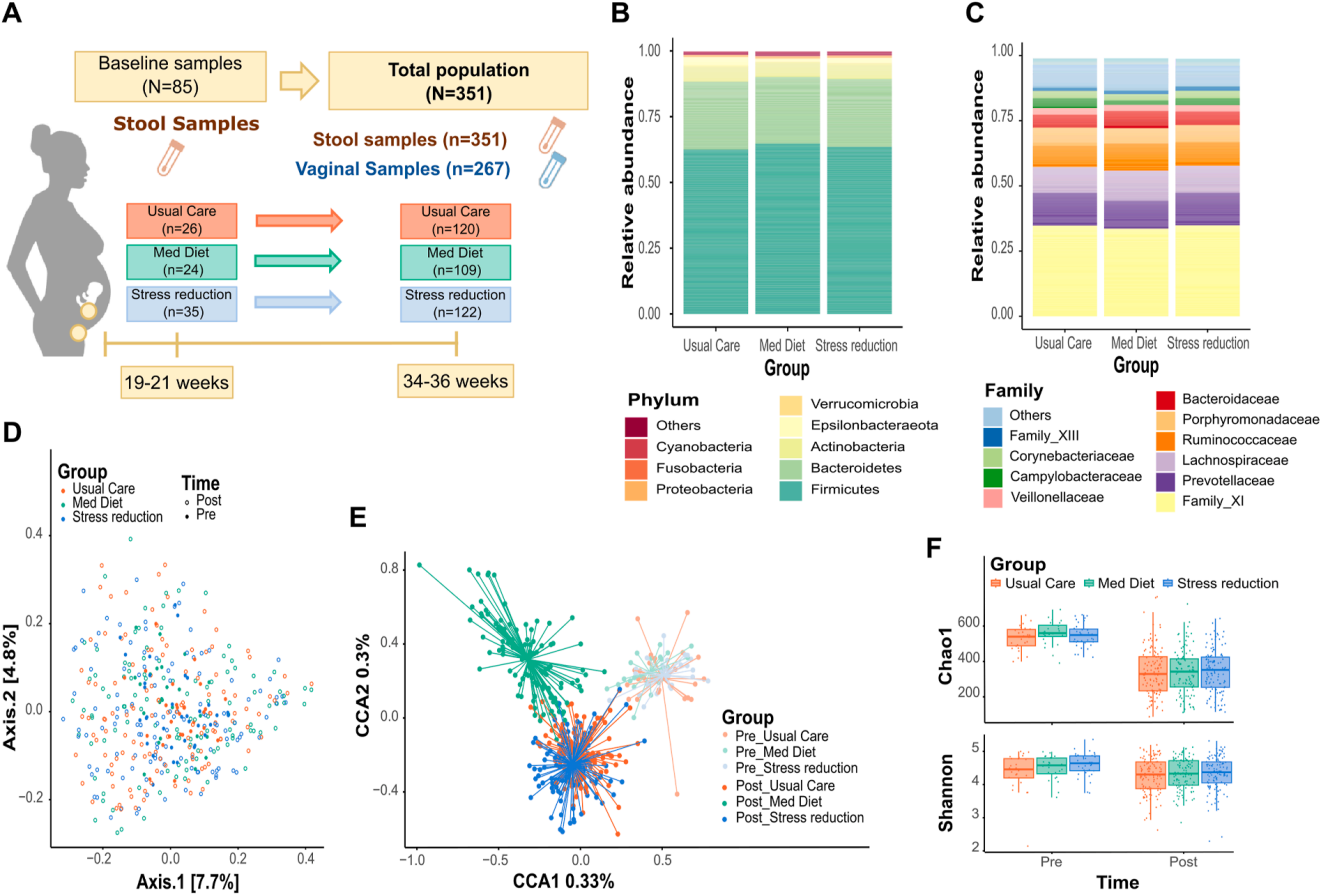
Association of the interventions with the composition and diversity of gut microbiota from pregnant individuals. (A) Diagram of the study design and the samples included in the study. (B) Bar plots showing microbiota profile of the fecal microbiota at phylum level according to group. Those phyla with a relative abundance of <1% across all samples were grouped as “Others.” (C) Bar plots showing the microbiota profile of the fecal microbiota of the main families (top 10 based on relative abundance) according to the interventional group. The rest of the families were grouped as “Others.” (D) Principal coordinate analysis of the mothers according to microbiota beta diversity based on the Bray-Curtis distance. The significance of the link between the intervention and the overall structure of gut microbiome was assessed by the Adonis test. (E) Canonical coordinate analysis of the pregnant individuals according to gestational intervention. (F) Influence of the gestational interventions on the alpha diversity of the gut microbiota measured as Chao1 and Shannon index. Comparisons between the interventional groups were assessed by the Kruskal-Wallis test after distribution testing.

Data of participants included in the study were pseudoanonymized and entered an electronic case report form. All individuals included in the trial had a baseline visit (19–23 wk) and a final visit (34–36 wk) with a dietitian to assess a validated 151-item food frequency questionnaire [12], and the validated pregnancy-adapted Mediterranean Diet Adherence Screener (preg-MEDAS) score validated for gestation (ATOT index; score range: 0 to 17 [13]. All participants also provided self-report lifestyle questionnaires to measure their anxiety and mindful state (State-trait Anxiety Inventory-STAI Anxiety and Personality; Perceived Stress Scale-PSS; WHO Five Well Being Index-WHO5; Five Facet Mindfulness Questionnaire-FFMQ)*.* During the last evaluation during gestation (34–36 wk), maternal fecal and vaginal samples were also collected and stored at −80°C until further analysis. As mentioned, in a subset of the participants (*n* = 85), fecal samples were also collected at the baseline visit (Figure 1A). Perinatal results were recorded in the hospital database, and medical discharge was within 28 d after delivery.

### Maternal fecal and vaginal sample collection and DNA extraction

Pregnant individuals' fecal and vaginal samples were collected at 34–36 wk of gestation for all the participants (*N* = 351) and at 19–23 wk of pregnancy or those with baseline data (*n* = 85), using a sterile cotton-tipped swab and stored at −80°C until analysis. All fecal and vaginal swab DNA was isolated using the MasterPure Complete DNA & RNA Purification Kit (Epicentre) according to the manufacturer's instructions with modifications that included a bead-beater step and enzyme incubation to increase DNA extraction as described elsewhere [8].

### Microbiota profiling and statistical analysis

For the taxonomical profiling of the pregnant individuals’ microbiota, the region V3-V4 from the 16S rRNA gene was sequenced using Illumina technology (Nextera XT Index Kit) using the primers specified elsewhere [14]. Amplicons were checked with a Bioanalyzer DNA 1000 chip, and libraries were sequenced using a 2 × 300 pb paired-end run (MiSeq Reagent kit v3) on a MiSeq-Illumina platform (FISABIO sequencing service). During DNA extraction and PCR amplification, controls were included and sequenced. The gut microbiome of the subset of individuals (*n* = 85) with longitudinal sampling was assessed by sequencing of the same region but through 2 × 250 pb paired-reads run on a NovaSeq-Illumina platform (Novogen) for both collection time points. Computational analysis of the resulting amplicons was performed using RStudio v 2023.6.0.421 software (Posit team, 2023. RStudio: Integrated Development Environment for R. Posit Software, PBC) and specifically, dada2 v.1.22.0 package [15] in both fecal datasets and vaginal samples, independently.

Clinical data are presented as mean (SD or SE), median (interquartile range [IQR]), or number (percentage), as appropriate. Statistical analysis for comparison of clinical and perinatal characteristics included the use of ANOVA or ANCOVA with baseline adjustment for continuous variables and a χ^2^ test for categorical variables. Within- and between-group differences were expressed as estimated means and 95% confidence interval (CI). In Supplemental material, for changes in foods and nutrient’s intake data analysis, we included only participants with available FFQ at baseline and final visit (*N* = 314). Analyses were performed using SPSS v. 27.0 (SPSS Inc.). Differences were considered significant when the *P* value < 0.05. Extended descriptions of the computational and statistical methods used for microbiota composition and diversity analysis are specified in Supplemental data (Materials and Methods).

## Results

### Characteristics of the study population

Baseline and perinatal characteristics of the study populations are shown in TABLE 1, TABLE 2, with no relevant differences observed among the study groups. Although participants in the three study groups reported similar baseline patterns of food and nutrient intake, at final assessment, significant differences were observed (see Supplemental Tables S1 and S2), and final preg-MEDAS score significantly increased for the Mediterranean diet group (Supplemental Table S1). Similarly, although no differences were reported at recruitment, participants in the Stress Reduction group had significantly higher scores on the mindful state-related questionnaire at the end of the intervention (Supplemental Table S3).

**TABLE 1:** Female characteristics of the study population in the intervention (*n* = 351).

Characteristics	Usual care	Mediterranean diet	Stress reduction	*P* value
	*n* = 120	*n* = 109	*n* = 122	
Age at recruitment (y)	36.7 (33.2–40.5)	37.3 (34.9–40.7)	38.3 (35.4–40.7)	0.113
Ethnicity, *n* (%)				
White	98 (81.7)	88 (80.7)	103 (84.4)	0.742
Latin	15 (12.5)	11 (10.1)	15 (12.3)	0.823
Afro-American	3 (2.5)	4 (3.7)	1 (0.8)	0.343
Asian	2 (1.7)	3 (2.8)	1 (0.8)	0.527
Others	2 (1.7)	3 (2.8)	2 (1.6)	0.793
Socio-economic status,^1^*n* (%)				
Low	6 (5)	7 (6.4)	5 (4.1)	0.724
Medium	42 (35.0)	42 (38.5)	34 (27.9)	0.231
High	72 (60.0)	60 (55)	83 (68)	0.122
BMI before pregnancy (kg/m^2^)	23.3 (4.8)	24.6 (5.2)	23.6 (4.1)	0.115
Medical history, *n* (%)				
Autoimmune disease	23 (19.2)	9 (8.3)	19 (15.6)	0.060
Thyroid disorders	12 (10)	10 (9.2)	16 (13.1)	0.590
Obesity (BMI ≥30)	11 (9.2)	15 (13.8)	10 (8.2)	0.338
Psychiatric disorders	6 (5)	4 (3.7)	10 (8.2)	0.307
Chronic hypertension	4 (3.3)	5 (4.6)	2 (1.6)	0.433
Chronic kidney disease	2 (1.7)	3 (2.8)	1 (1.6)	0.793
Obstetric history, *n* (%)				
Nulliparous	52 (43.3)	55 (50.5)	41 (33.6)	0.033
Previous SGA or preeclampsia	26 (21.7)	21 (19.3)	14 (11.5)	0.092
Previous preterm birth	7 (5.8)	9 (8.3)	4 (3.3)	0.264
Use of assisted reproductive technologies	30 (25)	22 (20.2)	40 (32.8)	0.088
Cigarette smoking during pregnancy	5 (4.2)	9 (8.3)	5 (4.1)	0.287
Alcohol intake during pregnancy	4 (3.3)	3 (2.8)	2 (1.6)	0.192
Drugs consumption during pregnancy	1 (0.8)	0 (0)	0 (0)	0.645
Sports practice during pregnancy	30 (25)	21 (19.3)	35 (28.9)	0.119
Yoga or Pilates during pregnancy	27 (22.5)	17 (15.6)	24 (19.7)	0.416

SGA, Small for gestational age. Categorical variables (Ethnicity, Socio-Economic status, medical and obstetric history, etc., are expressed as number of positive cases and percentage.

Data are expressed as median (IQR), mean (SD), or *n* (%).

1 Socioeconomical status: low (never worked or unemployed >2 y), medium (secondary studies and work), high (university studies and work)

**TABLE 2:** Pregnancy and perinatal outcome of the study population according to intervention groups (*n* = 351).

Characteristics	Usual care	Mediterranean diet	Stress reduction	*P* value
	*N* = 120	*N* = 109	*N* = 122	
Gestational age at recruitment (wk)	20.8 (0.7)	20.8 (0.6)	20.9 (0.8)	0.086
Treatment during pregnancy, *n* (%)				
Aspirin	31 (25.8%)	41 (37.6%)	41 (33.6%)	0.149
Heparin	5 (4.2%)	10 (9.2%)	7 (5.7%)	0.283
Antihypertensive drugs	2 (1.7%)	3 (2.8%)	2 (1.6%)	0.793
Progesterone	12 (10%)	9 (8.3%)	14 (11.5%)	0.717
Steroids	1 (0.8%)	4 (3.7%)	7 (5.7%)	0.109
Magnesium sulfate	1 (0.8%)	2 (1.8%)	2 (1.6%)	0.791
Iron	50 (41.7%)	42 (38.5%)	60 (49.2%)	0.240
Levothyroxine	10 (8.3%)	9 (8.3%)	19 (15.6%)	0.113
Antibiotics	11 (9.2%)	7 (6.4%)	18 (14.8%)	0.101
Antifungal	7 (5.8%)	5 (4.6%)	7 (5.7%)	0.900
Pregnancy complications, *n* (%)				
Preeclampsia	10 (8.3%)	8 (7.3%)	5 (4.1%)	0.382
Prenatally diagnosed SGA	13 (10.8%)	7 (6.4%)	11 (9.1%)	0.488
Prenatally diagnosed severe SGA	8 (6.7%)	4 (3.7%)	4 (3.3%)	0.780
Delivery outcome, *n* (%)				
Gestational age at delivery (wk)	39.6 (1.3)	39.7 (1.3)	39.6 (1.4)	0.836
Preterm birth	3 (2.5%)	5 (4.6%)	7 (5.7%)	0.452
Induction of labor	67 (55.8%)	55 (50.5%)	66 (54.1%)	0.665
Mode of delivery				
Vaginal delivery	66 (55%)	49 (45%)	77 (63.1%)	0.017
Operative vaginal delivery	14 (11.7%)	16 (14.7%)	10 (8.3%)	0.310
Cesarean section	39 (32.5%)	44 (40.5%)	34 (27.9%)	0.141
Maternal anesthesia	107 (89.2%)	97 (89%)	108 (88.5%)	0.567
Antibiotics during labor	56 (46.7%)	56 (51.4%)	59 (48.4%)	0.836
Neonatal outcome, *n* (%)				
Female	50 (41.7%)	55 (50.5%)	54 (44.3%)	0.428
Birthweight (g)	3285 (2844-3594)	3230 (2984-3515)	3300 (2980-3589)	0.920
Birthweight (percentile)	43 (30.4)	42.3 (30.9)	41 (26.6)	0.830
Small for gestational age	24 (20%)	19 (17.4%)	17 (13.9%)	0.453
pH umbilical artery	7.21 (0.1)	7.20 (0.1)	7.21 (0.1)	0.975
Neonatal complications	9 (7.5%)	11 (10.1%)	7 (5.7%)	0.460
NICU admission	6 (5%)	3 (2.8%)	4 (3.3%)	0.631

NICU, neonatal intensive care unit; PE, preeclampsia; SGA, small for gestational age. Categorical variables are expressed as number of positive cases. Data are expressed as median (IQR), mean (SD), or *n* (%).

### Relation of lifestyle interventions to maternal gut microbiota

Overall, gut microbiota from pregnant individuals at 34–36 wk was dominated by Firmicutes (mean: 63.43%; *Peptoniphilus* the most relatively abundant genus, median: 9.62%), followed by Bacteroidetes (mean: 26.31%, *Porphyromonas* as the main representative genus, mean: 5.38%) and Actinobacteria (mean: 5.14%, being *Corynebacterium* its more represented genus, median: 1.06%) (Figure 1B and C).

At the end of the intervention, analysis based on Bray-Curtis distance revealed a slight influence of the intervention (R2 = 0.009, F = 1.56, *P* = 0.005) (Figure 1D) on the overall structure of the pregnant individuals' gut microbiota, with pregestational BMI (BMI, kg/m^2^) being a significant factor modulating the microbiota profile (R2 = 0.006, F = 2.25, *P* = 0.001). Furthermore, interventions modulated the microbiota composition, as shown in the canonical coordinate analysis (CCA model; F = 1.218, *P* = 0.001) (Figure 1E). Regarding alpha diversity, no differences were observed in microbial diversity (Kruskal-Wallis test Shannon index, *P* = 0.583) or richness (Kruskal-Wallis test Observed ASV, *P* = 0.761) according to the interventional groups (Figure 1F). However, results revealed a decrease in both alpha diversity (Kruskal-Wallis test Shannon index, *P* < 0.001) and richness (Kruskal-Wallis test Observed ASV, *P* < 0.001) as pregnancy progressed (pre- compared with postsamples). However, this decrease in alpha diversity is more observable in those mothers who followed one of the interventions. In general, results revealed that both alpha and beta diversity of the pregnant individuals' gut microbiota showed higher interpersonal variance throughout the pregnancy, as can be observed in the increment in the dispersion in the post- compared with presamples (Figure 1D and E; Supplemental Figure S1).

Furthermore, in a subset of samples with baseline data (*n* = 85), overall structure of the gut microbiota was similar among groups before the intervention (Adonis test on Bray-Curtis index; R2 = 0.027, *P* = 0.184; presamples), whereas significant differences were observed between intervention groups after it in beta diversity models (Adonis; R2 = 0.033, *P* = 0.011; Post samples) and at composition level (CCA model; F = 1.170, *P* = 0.001) (Supplemental Figure S1). Due to the differential nature of both interventions, the potential impact of both dietary and Stress reduction programs on microbiota modulation was assessed independently as described below.

### Maternal gut microbiota and Mediterranean diet intervention

**FIGURE 2:**
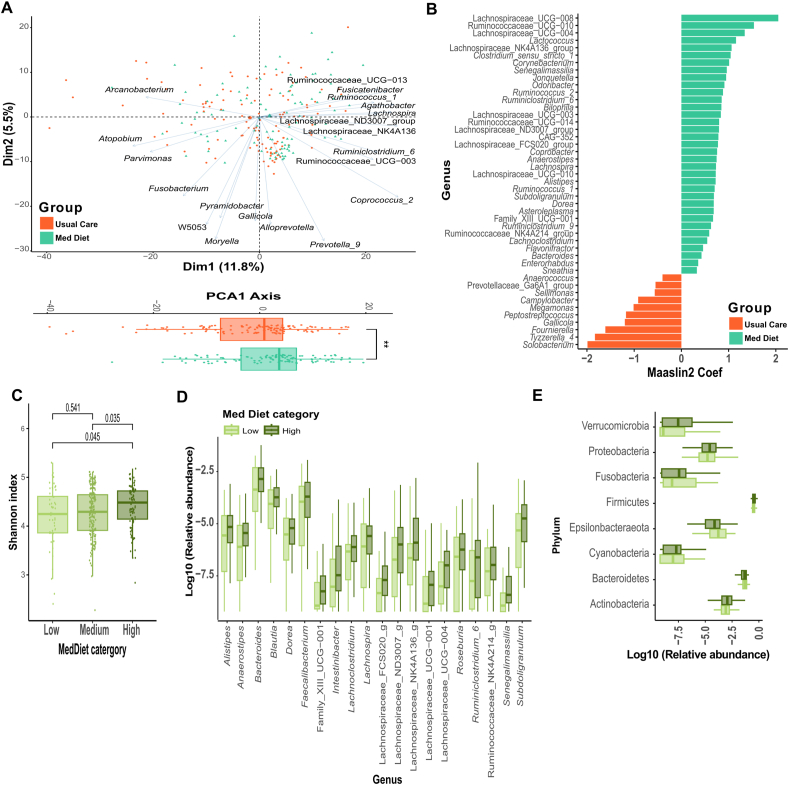
Impact of the dietary intervention based on a Mediterranean diet on the gut microbiota of pregnant individuals. (A) Biplot of the PCA of the fecal microbiota at genus level and the most relevant genera that contribute to each component distribution (*n* = 20). Data were transformed using a centered-log ratio. Boxplot showing the distribution of the samples on the PCA1 component based on the fecal microbiota composition. Statistical difference between component values of the groups was assessed by the Wilcoxon rank-sum test. (B) Multivariate analysis (Maaslin2) modeling the effect of the Mediterranean diet intervention on the gut microbiome compared with the usual care group at genus level. Body mass index and antibiotic use are included as covariates. Only those genera with q-values <0.2 were included in the plot. (C) Alpha diversity measured as Shannon index based on the adherence to the Mediterranean diet in the whole population (*n* = 351). Mothers were classified based on the score from the 17-item Mediterranean adherence assessment questionnaire as having high (Med diet score >11), median (Med diet score, 6–11), and low adherence (Med diet score <6). Comparisons between the adherence groups were assessed by the Kruskal-Wallis test after distribution testing with Dunn’s post-hoc test and for multiple comparisons adjustment. (D-E) Differences at genus (D) and phylum (E) level between the Mediterranean diet adherence groups determined by Maaslin2 linear models adjusted by prepregnancy BMI and antibiotic use. Only mothers classified as High (*N* = 98) and Low (*N* = 53) adherence were included in the models. All phyla with a prevalence >70% are represented, and only those genera with the same prevalence threshold and q-value < 0.1 are plotted. Complete results of the linear models are available in Supplemental Table S4 along with the coefficients, the 95% confidence intervals, and the SEs.

Mediterranean diet-based counseling intervention had a significant influence on the overall structure of the gut microbiota at the end of the intervention (Adonis test on the Bray-Curtis distance; R2 = 0.008, F = 1.885, *P* = 0.002). Principal component analysis (PCA) showed a separation between the samples according to the intervention, with a significant distribution of the samples (x-axis distribution *P* = 0.004; Figure 2A). Indeed, some genera were overrepresented in these pregnant individuals compared with those without intervention. Multivariate analysis was performed to decipher the microbial markers representative of each group (Figure 2B). At the phylum level, the dietary intervention was characterized by a decrease in the relative abundance of Epsilonbacteraeota phylum (Coeff = −0.92; 95% CI: −1.37, −0.46; q = 0.001) compared with the Usual Care group (Table 3). At genus level, specific enrichment of SCFA producers’ genera such as *Ruminococcaceae* and *Lachnospiraceae* families were found in mothers from the Mediterranean diet group (Figure 2B; Table 3), as other genera such as *Bacteroides* (Coeff = 0.43; 95% CI: 0, 0.86; q = 0.175), *Odoribacte* (Coeff = 0.91; 95% CI: 0.32, 1.51; q = 0.018), or *Dorea* (Coeff = 0.69; 95% CI: 0.08, 1.29; q = 0.108) genera. On the contrary, microbiota from pregnant individuals who followed a usual care program was overrepresented by *Campylobacter* (Coeff = −0.91; 95% CI: −1.37, −0.46; q = 0.001), *Anaerococcus* (Coeff = −0.4; 95% CI: −0.77, −0.03; q = 0.132), and *Gallicola* (Coeff = −1.2; 95% CI: −2.2, −0.2; q = 0.087) genera. No effect of the intervention was observed in the alpha diversity and richness of the female’s gut microbiota (Figure 1E).

**TABLE 3:** Multivariable model for the gut microbiota composition at phylum and genus level from pregnant individuals who followed the Mediterranean diet intervention (*N* = 109) compared with usual care (*N* = 120).

	Coef	95% CI	SE	Prev.	*P* value	Q value
Phylum						
Epsilonbacteraeota	-0.92	(−1.37, −0.46)	0.23	99.13	<0.001	0.001
Firmicutes	0.06	(0, 0.12)	0.03	100	0.044	0.161
Proteobacteria	0.39	(−0.01, 0.8)	0.21	99.13	0.059	0.198
Tenericutes	1.13	(0.5, 1.76)	0.32	69.87	0.001	0.003
Genus						
*Alistipes*	0.73	(0.14, 1.32)	0.3	97.82	0.017	0.075
*Anaerococcus*	−0.4	(−0.77, −0.03)	0.19	99.56	0.036	0.132
*Anaerostipes*	0.75	(0.21, 1.28)	0.27	98.25	0.007	0.037
*Asteroleplasma*	0.68	(0.25, 1.12)	0.22	59.39	0.002	0.014
*Bacteroides*	0.43	(0, 0.86)	0.22	100	0.053	0.175
*Bilophila*	0.85	(0.16, 1.53)	0.35	74.67	0.016	0.073
CAG-352	0.78	(0.07, 1.49)	0.36	43.23	0.031	0.120
*Campylobacter*	−0.91	(−1.37, −0.46)	0.23	99.13	<0.001	0.001
*Clostridium_sensu_stricto_1*	1.04	(0.19, 1.9)	0.44	77.73	0.018	0.077
*Coprobacter*	0.76	(0.12, 1.39)	0.32	52.4	0.02	0.087
*Corynebacterium*	1.01	(0.23, 1.79)	0.4	53.71	0.012	0.057
*Dorea*	0.69	(0.08, 1.29)	0.31	98.25	0.027	0.108
*Enterorhabdus*	0.36	(0.01, 0.7)	0.18	29.69	0.045	0.157
Family_XIII_UCG-001	0.67	(0.06, 1.28)	0.31	89.52	0.033	0.124
*Fournierella*	−1.61	(−2.11, −1.11)	0.25	38.43	<0.001	<0.001
*Gallicola*	−1.2	(−2.2, −0.2)	0.51	54.59	0.020	0.087
*Jonquetella*	0.95	(0.25, 1.64)	0.36	41.05	0.008	0.042
*Lachnoclostridium*	0.55	(−0.02, 1.11)	0.29	95.2	0.058	0.188
*Lachnospira*	0.73	(0.03, 1.43)	0.36	95.63	0.041	0.145
Lachnospiraceae_FCS020_group	0.77	(0.2, 1.35)	0.29	86.03	0.009	0.046
Lachnospiraceae_ND3007_group	0.8	(0.06, 1.54)	0.38	96.51	0.036	0.133
Lachnospiraceae_NK4A136_group	1.06	(0.3, 1.83)	0.39	95.63	0.007	0.038
Lachnospiraceae_UCG-003	0.84	(0.34, 1.33)	0.25	82.97	0.001	0.008
Lachnospiraceae_UCG-004	1.34	(0.75, 1.93)	0.3	92.14	<0.001	<0.001
Lachnospiraceae_UCG-008	2.05	(1.55, 2.56)	0.26	45.85	<0.001	<0.001
Lachnospiraceae_UCG-010	0.73	(0.17, 1.28)	0.28	37.99	0.011	0.052
Lactococcus	1.16	(0.54, 1.77)	0.31	70.74	<0.001	0.002
*Megamonas*	−1.01	(−1.36, −0.66)	0.18	18.34	<0.001	<0.001
*Odoribacter*	0.91	(0.32, 1.51)	0.3	89.96	0.003	0.018
*Peptostreptococcus*	−1.17	(−2.21, −0.14)	0.53	93.89	0.028	0.109
Prevotellaceae_Ga6A1_group	−0.55	(−0.93, −0.17)	0.19	54.15	0.005	0.029
*Ruminiclostridium_6*	0.85	(0.03, 1.67)	0.42	86.03	0.042	0.149
*Ruminiclostridium_9*	0.63	(−0.01, 1.27)	0.33	87.34	0.057	0.186
Ruminococcaceae_NK4A214_group	0.59	(0.07, 1.11)	0.27	95.2	0.028	0.110
Ruminococcaceae_UCG-010	1.54	(0.91, 2.17)	0.32	50.66	<0.001	<0.001
Ruminococcaceae_UCG-014	0.8	(0.2, 1.4)	0.31	93.45	0.010	0.048
*Ruminococcus_2*	0.88	(0.04, 1.72)	0.43	93.45	0.041	0.145
*Sellimonas*	−0.56	(−1.1, −0.03)	0.27	32.75	0.040	0.143
*Senegalimassilia*	0.96	(0.31, 1.62)	0.33	83.84	0.004	0.026
*Sneathia*	0.33	(0.03, 0.63)	0.15	20.96	0.034	0.128
*Solobacterium*	−1.99	(−2.76, −1.21)	0.4	42.79	<0.001	<0.001
*Subdoligranulum*	0.69	(0.03, 1.35)	0.33	97.82	0.041	0.145
*Tyzzerella_4*	−1.83	(−2.64, −1.02)	0.41	60.7	<0.001	<0.001

Multivariable analysis was performed using the Maaslin2 algorithm with the total sum scaling (TSS) normalization and log transformation. Prepregnancy BMI and antibiotics were also included in the model as potential confounding factors. Only those taxa with an FDR (q-value) <0.2 are shown in the table. Coeff: Maaslin2 Coefficient, 95% CI, 95% confidence intervals, Prev., Prevalence of the taxa in the total population.

Due to the significant effect of the dietary intervention, we wanted to explore in depth the potential impact of the Mediterranean diet on the gut microbiota of the pregnant population, independently of the group, and decipher the food groups and nutrients associated with this effect. When the general population was considered, mothers who were classified as having high adherence to the Mediterranean diet (ATOT index >11) showed a higher Shannon index than those classified as medium (Kruskal-Wallis test, *P* = 0.035) and low adherence (ATOT index < 6; Kruskal-Wallis test, *P* = 0.045) (Figure 2C). Mothers classified as high adherence to the Mediterranean diet reproduced similar biomarkers in their gut microbiota to those who followed a Mediterranean diet-based intervention, including the diminishment of Epsilonbacteraeota (mainly due to *Campylobacter* genus) phylum and enrichment in SCFA producers genera (Figure 2D and E, Supplemental Table S4), confirming the link between the intervention during pregnancy and the gut microbiome.

Then, we explored the associations of microbial taxa with specific food groups of the maternal diet during pregnancy. Results presented as a heatmap are reported in Supplemental Figure S2. Interestingly, the Mediterranean diet adherence index and food group that define the Mediterranean diet, such as fish, nuts, and legumes followed the same pattern of relations with significant associations with the microbial biomarkers of both the interventional group and the Mediterranean diet adherence in the whole population, including *Lachnospiraceae* and *Ruminoccocaceae* species, *Bacteroides* or *Odoribacter* being these genera negatively associated with the consumption of ultraprocessed products (Supplemental Figure S2).

### Maternal gut microbiota and stress reduction intervention

**FIGURE 3:**
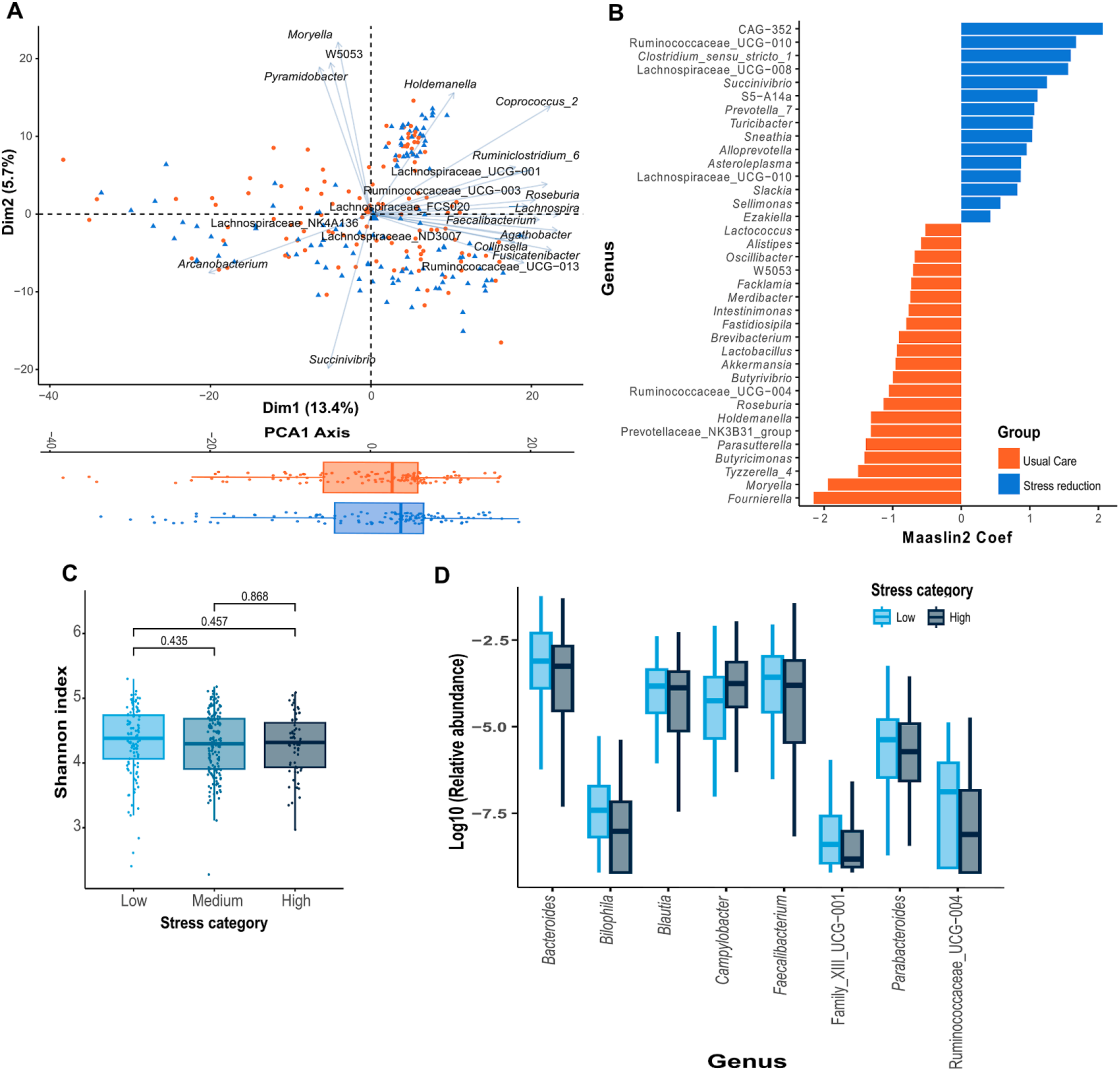
Impact of the Stress reduction intervention based on mindfulness sessions on gut microbiota of pregnant individuals. (A) Biplot of the PCA of the fecal microbiota at genus level and the most relevant genera that contribute to each component distribution (*n* = 20). Data were transformed using a centered-log ratio. Boxplot showing the distribution of the samples on the PCA1 component based on the fecal microbiota composition. Statistical difference between component values of the groups was assessed by the Wilcoxon rank-sum test. (B) Multivariate analysis (Maaslin2) modeling the effect of the Stress reduction intervention (*N* = 122) on the gut microbiome compared with the usual care group (*N* = 120) at genus level. Body mass index and antibiotic use are included as covariates. Only those genera with q-values <0.2 were included in the plot. (C) Alpha diversity measured as Shannon index based on the Stress category in the whole population (*n* = 351). Mothers were classified based on the score from the 5-well-being questionnaire as having high (5-WHO index <55), median (5-WHO index, 55–75), and low stress (5-WHO index >75). Comparisons between the stress groups were assessed by the Kruskal-Wallis test after distribution testing with Dunn’s post-hoc test and for multiple comparisons adjustment. (D) Differences at genus level between the stress groups determined by Maaslin2 linear models adjusted by prepregnancy BMI (kg/m^2^) and antibiotic use (yes/no). Only mothers classified as High (*N* = 60) and Low stress (*N* = 109) were included in the models. Only those genera with a higher prevalence than 70% and q-value ≤0.2 are plotted. Complete results of the linear models are available in Supplemental Table S5 along with the coefficients, the 95% confidence intervals, and the SEs.

Contrary to the dietary intervention, the Stress reduction program did not show a significant impact on the beta diversity of the gut microbiota (R2 = 0.005, F = 1.251, *P =* 0.094*;* Adonis test on the Bray-Curtis distance) (Figure 3A) or alpha diversity index (Figure 1E).

Although this showed that the strategy did not have a deep modulation on the gut microbiota of pregnant individuals, we found some associations with specific microbial components. Adjusted models revealed a higher representation of health-associated taxa including Ruminococcaceae_UCG-010 (Coeff = 1.68; 95% CI: 1.13, 2.22; q ≤ 0.001)*, Succinivibio* (Coeff = 1.25; 95% CI: 0.86, 1.64; q ≤ 0.001)*,* or *Turicibacter* (Coeff = 1.05; 95% CI: 0.19, 1.9; q = 0.066) genera (Figure 3B, Table 4) compared with the Usual care protocol.

**TABLE 4:** Multivariable model for the gut microbiota composition at phylum and genus level from pregnant individuals that followed the Stress reduction intervention (*N* = 122) compared with usual care (*N* = 120).

	Coef	95 CI	SE	Prev.	*P* value	q value
Phylum						
Verrucomicrobia	−0.96	(−1.86, −0.06)	0.46	73.55	0.038	0.154
Genus						
*Akkermansia*	−0.96	(−1.86, −0.05)	0.46	73.55	0.039	0.128
*Alloprevotella*	0.96	(0.39, 1.52)	0.29	65.7	0.001	0.007
*Asteroleplasma*	0.87	(0.42, 1.32)	0.23	57.85	<0.001	0.002
*Brevibacterium*	−0.91	(−1.79, −0.03)	0.45	50.41	0.043	0.139
*Butyricimonas*	−1.41	(−1.77, −1.05)	0.18	42.15	<0.001	<0.001
*Butyrivibrio*	−1	(−1.76, −0.24)	0.39	29.34	0.011	0.048
CAG-352	2.06	(1.39, 2.74)	0.35	61.98	<0.001	<0.001
*Clostridium_sensu_stricto_1*	1.6	(0.77, 2.42)	0.42	84.71	<0.001	0.002
*Ezakiella*	0.43	(−0.01, 0.87)	0.22	99.59	0.060	0.171
*Fastidiosipila*	−0.8	(−1.55, −0.05)	0.38	89.67	0.037	0.125
*Fournierella*	−2.15	(−2.57, −1.73)	0.21	31.4	<0.001	<0.001
*Holdemanella*	−1.32	(−2.34, −0.29)	0.52	47.52	0.012	0.054
Lachnospiraceae_UCG-008	1.56	(1, 2.12)	0.29	36.36	<0.001	<0.001
Lachnospiraceae_UCG-010	0.87	(0.24, 1.5)	0.32	36.36	0.008	0.035
*Merdibacter*	−0.74	(−1.44, −0.05)	0.35	64.05	0.037	0.125
*Moryella*	−1.94	(−2.88, −1.01)	0.48	61.16	<0.001	0.001
*Oscillibacter*	−0.68	(−1.37, 0.01)	0.35	73.14	0.056	0.166
*Parasutterella*	−1.39	(−2.12, −0.67)	0.37	91.74	<0.001	0.002
*Prevotella_7*	1.07	(0.2, 1.93)	0.44	76.03	0.016	0.065
Prevotellaceae_NK3B31	−1.32	(−2.09, −0.55)	0.39	78.51	0.001	0.007
*Roseburia*	−1.13	(−1.91, −0.36)	0.4	80.58	0.005	0.024
Ruminococcaceae_UCG-004	−1.06	(−2.08, −0.03)	0.52	71.9	0.045	0.140
Ruminococcaceae_UCG-010	1.68	(1.13, 2.22)	0.28	62.81	<0.001	<0.001
S5-A14a	1.11	(0.53, 1.69)	0.3	98.76	<0.001	0.002
*Sellimonas*	0.57	(0.06, 1.08)	0.26	60.74	0.028	0.101
*Slackia*	0.82	(0.16, 1.48)	0.34	83.06	0.016	0.065
*Sneathia*	1.03	(0.67, 1.4)	0.19	35.12	<0.001	<0.001
*Succinivibrio*	1.25	(0.86, 1.64)	0.2	36.36	<0.001	<0.001
*Turicibacter*	1.05	(0.19, 1.9)	0.44	64.46	0.017	0.066
*Tyzzerella_4*	−1.51	(−2.39, −0.62)	0.45	60.74	0.001	0.007
W5053	−0.7	(−1.24, −0.16)	0.28	28.51	0.011	0.050

Multivariable analysis was performed using the Maaslin2 algorithm with the total sum scaling (TSS) normalization and log transformation. Prepregnancy BMI (kg/m^2^) and antibiotics were also included in the model as potential confounding factors. Only those taxa with an FDR (q-value) <0.2 are shown in the table. Coeff: Maaslin2 Coefficient; 95% CI, 95% confidence intervals; Prev., Prevalence of the taxa in the total population.

As we did for the Mediterranean diet adherence, we evaluated the influence of stress during pregnancy, assessed by the well-being questionnaire, on the whole population (Figure 3C and D). We found that mothers classified in the low stress group (WHO-5 index >75) showed a gut microbiota enriched in health-associated taxa, such as Ruminococcaceae_UCG-010 (Coeff = −0.79; 95% CI: −1.58, 0; q = 0.184), *Faecalibacterium* (Coeff = −0.81; 95 % CI: −1.54, −0.06; q = 0.144), *Blautia* (Coeff = −0.63; 95% CI: −1.23; −0.02; q = 0.169), and Ruminococcaceae_UCG-004 (Coeff = −1.35; 95% CI: −2.5, −0.18; q = 0.120), among others; and a diminishment in *Campylobacter* genus (Coeff = 0.67; 95% CI: 0.16, 1.18; q = 0.069), compared with those classified as high-stressed mothers (WHO-5 index < 55) (Figure 3D, Supplemental Table S5). No association was observed between the stress category and the alpha diversity of the gut microbiota (Figure 3C).

### Lifestyle interventions and the vaginal microbiota of pregnant individuals

**FIGURE 4:**
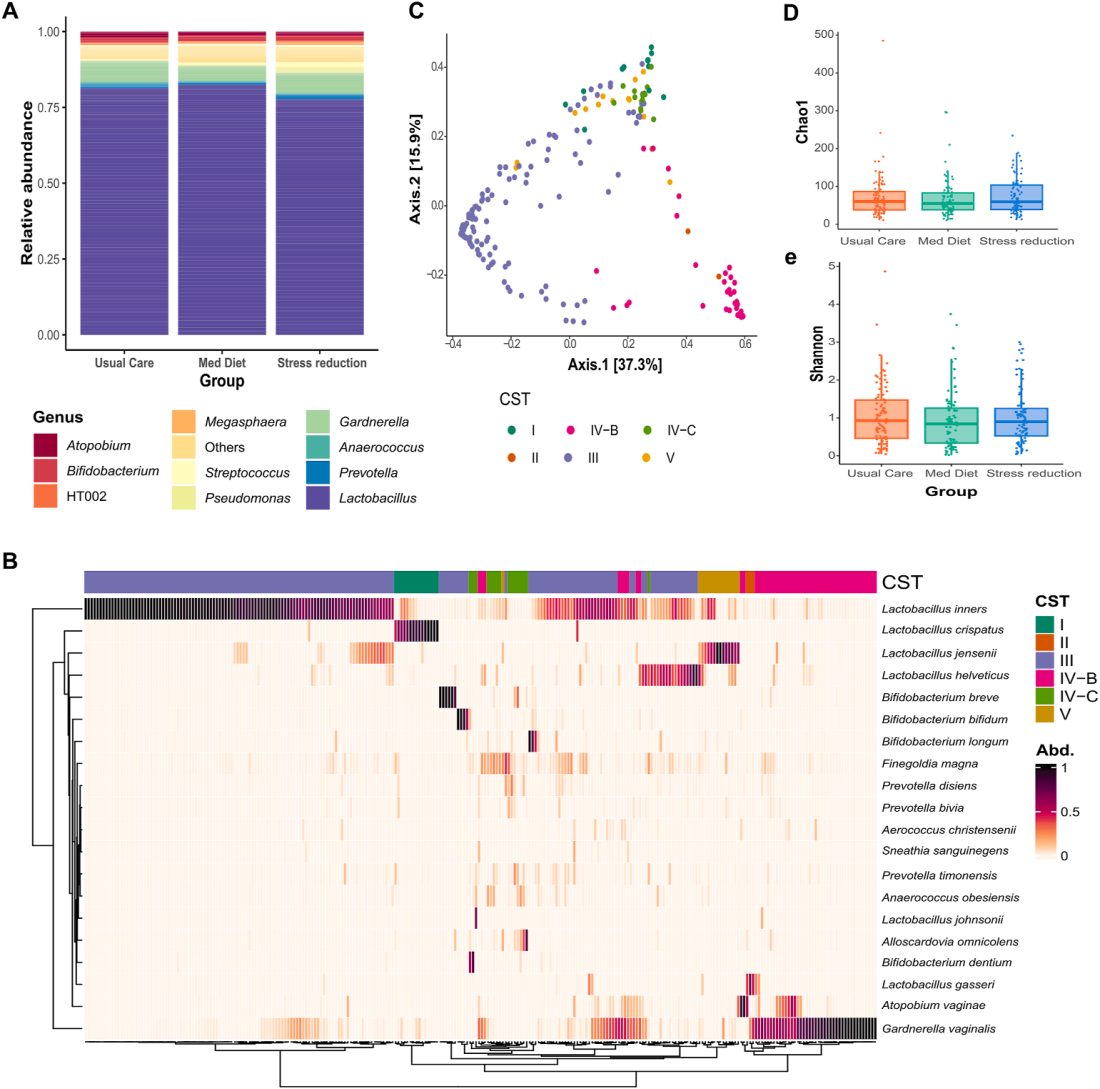
Relation of vaginal microbiota of pregnant individuals to gestational intervention. (A) Bar plots showing microbiota profile of the vaginal microbiota at genus level according to group. The top 10 taxa are shown with the rest grouped as “Others.” (B) Heatmap showing the distribution of the relative abundance from the top 20 species on the vaginal microbiota of pregnant individuals. Color legend shows the community state type (CST) assessed by the VALENCIA tool (see methods). (C) Principal coordinate analysis of the mothers according to microbiota beta diversity based on the Bray-Curtis distance. The significance of the link between the intervention and CST was assessed by the Adonis test. (D) Influence of the gestational interventions on the alpha diversity of the vaginal microbiota measured as Observed ASV, Chao1, and Shannon index. Comparisons between the interventional and the Usual Care groups were assessed by the Kruskal-Wallis test after distribution testing.

Vaginal microbiota was mainly characterized by the presence of *Lactobacillus* genus (median = 93.7%, IQR = 25.3) followed by *Prevotella* (median: 49.4%, IQR = 32.6) and *Anaerococcus* (median = 31.7%, IQR = 15.8) genera (Figure 4A). At species level, the samples showed a differential pattern of distribution based on the presence of different species (Figure 4B). The main group was clearly overrepresented by *Lactobacillus inner* and the other by *Gardnerella vaginalis*. This distribution was also observed in the organization of the samples in the beta diversity analysis based on Bray-Curtis distance (Figure 4C), which matched with the previously described community state types, which was observed as the main factor shaping the vaginal microbiota overall community (R2 = 0.148, F = 8.56, *P* = 0.001). In the multivariate analysis based on the Bray-Curtis distance, no significant impact of the intervention was found in the vaginal microbiota (R2 = 0.006, F = 0.89, *P* = 0.485). Similarly, no association between any intervention was observed and alpha diversity (Figure 4D).

## Discussion

### Principal findings of the study

This subanalysis of the IMPACT BCN trial demonstrates a significant relation between lifestyle interventions (Mediterranean diet or Stress reduction) during pregnancy and the composition of the gut microbiota of pregnant individuals. Dietary intervention showed a deeper impact on the overall structure of the gut microbiota, whereas Stress reduction strategy modulated the relative abundance of some key health-associated genera. Although the interventions had only a slight effect on the overall structure of the gut microbiome, they—especially the one based on the Mediterranean diet—affected key microbial members in the gut of pregnant individuals, highlighting their potential role in the beneficial effect of these interventions in reducing adverse gestational health outcomes.

### Maternal gut microbiota and Mediterranean diet intervention

Diet is one of the factors that most influence the microbiota [14] and is considered an easy-to-implement intervention targeting gut microbiota. In our study, the association between the Med Diet intervention and the gut microbiota overall structure was comparable to other well-recognized factors modulating its composition, such as the maternal body mass index. A growing body of evidence has shown that a Mediterranean diet could modulate the gut microbiota, increasing its diversity and changing the proportion of some bacteria, compared with a Western food model [16]. In our study, participants in the Mediterranean diet group showed a microbiota enriched in members of Ruminococcaceae and Lachnospiraceae families, *Bacteroides*, *Dorea,* and *Odoribacter* genera. *Odoribacter* was found to decrease in some metabolic disorders, such as obesity [17,18], and was affected by dietary interventions with low-fat diets [19].

Pregnant individuals’ microbiota at delivery has been reported to be clustered according to diet in previous studies [16]. *Blautia*, *Bifidobacterium*, and several groups from Ruminococcaceae and Lachnospiraceae families were associated with a high intake of plant foods and some types of fatty acids typically of the Mediterranean diet. In fact, we also found a positive association between these families and the consumption of some of the key nutrients in this dietary model, including legumes and blue fish. Both food groups are rich in nutrients that have been reported to influence gut microbiota composition, including fibers [20] and n–3 PUFAs from the legumes and fish, respectively. Interestingly, those genera associated with these food groups from the Mediterranean diet showed positive correlations with those that characterize mothers from the dietary intervention group, supporting the effect of the dietary counseling on the gut microbiota composition. Although the dietary counseling intervention did not impact the alpha diversity of the pregnant individuals’ microbiota, we confirm in our study population that adherence to a Mediterranean diet was associated with an increase in both the diversity of the gut bacterial community compared with those with low adherence to this dietary pattern. Similarly, several health-associated genera, such as *Blautia*, *Faecalibacterium*, or *Christensenella*, were increased in those mothers who follow a Mediterranean diet during gestation. These results are in line with previous studies on the general population following a Mediterranean diet and some studies on the pregnant population [21].

### Maternal gut microbiota and stress reduction intervention

Stress and well-being are also considered determinants of gut microbiota. During pregnancy, stressors have also been described to impact the gut microbial composition, and pregnant individuals exposed to prenatal stress had significantly different fecal microbial community composition than nonstressed females [22]. Although the relation between the dietary intervention and gut microbiota was deeper than the Stress reduction strategy, we observed a significant link between this intervention and some members of the maternal gut microbiota composition. Gut microbiota from Stress intervention pregnant individuals harbored a higher relative abundance of *Turicibacter* genera along with some Lachnospiraceae and Ruminococcaceae families, in agreement with those studies [23] that found a reduction of these taxa associated with some mental disorders in general population. A recent study demonstrated molecular mechanisms by which *Turicibacter* spp*.* could impact the fat metabolism in the host through bile acids transformations [24]. Although most studies have been performed on the general population, few studies have linked stressors and intestinal microbial composition during pregnancy [25]. Hechler et al. [7] found in a pregnant population that general perinatal stress indicators were negatively correlated with *Eubacterium* and *Oscillospira* genera, both associated with the capacity for butyrate production. We also found in the whole study population that high stress was associated with lower relative abundance of some SCFA producers, such as *Faecalibacterium* or Ruminococcaceae spp. To our knowledge, this is the first time that a structured stress reduction intervention shows an association with the microbial population of gut microbiota and opens the door to the use of gut biomarkers as outcomes in psychological intervention trials, in the framework of precision psychiatry and psychotherapy [26].

### Maternal vaginal microbiota

Usually, vaginal microbiota is considered a low-diversity environment dominated by *Lactobacillus* spp., and vaginal microbiota during gestation was described as less diverse with a higher predominance of *Lactobacillus* genus [27]. Previously, few studies explored the effect of dietary interventions targeting the vaginal microbiota. Houttu et al. [28] report a reduction in the relative abundance of some pathobionts, such as *Ureaplasma*, in a group of pregnant females with overweight after fish oil and/or prebiotic consumption [29]. Others described that a general healthy diet pattern and fiber intake or some micronutrient consumption, can reduce the risk of developing bacterial vaginosis [28]. However, most of these analyses were focused on the context of bacterial vaginosis. In our analysis, we found the previously described community state type in the maternal cohort; however, no association between the interventions and vaginal microbiota was observed.

### Strengths and limitations

As a strength, this study represents the first description from a randomized trial demonstrating the link between lifestyle interventions during pregnancy and gut microbiota. As a limitation, we acknowledge that microbiota was not the primary outcome of the trial. Also, the relatively small sample size prevented a more comprehensive analysis of the association of microbiota and specific perinatal outcomes in each study group. In addition, the lack of samples collected before starting the intervention for all the participants limited the analysis of longitudinal microbiota changes in each participant. Analyses were adjusted by maternal BMI and antibiotics use during pregnancy; however, we acknowledge that other potential confounders might have influenced the results. Finally, future studies are warranted to determine the influence of observed microbiota changes on perinatal and postnatal outcomes.

In conclusion, lifestyle interventions during pregnancy influence maternal gut microbiota, with a minimal association with vaginal microbiota. To our knowledge, this is the first study that provides evidence of the link between the Mediterranean diet and Stress reduction strategies and the maternal gut microbiota and highlights the maternal gut microbiota as a potential therapeutic target. Future studies are warranted to assess the potential long-term benefits of microbiota as a therapeutic target.

## Authors contributions

The author’s responsibilities were as follows – M.S-R: Formal analysis, data curation, data analysis, writing – original draft, visualization; FC: Conceptualization, methodology, validation, writing – original draft, supervision, project administration; S.C-B: Investigation on Mediterranean diet intervention; IC, M.L, MG, CP, LB, LY, RP, NE, AN: Investigation; AA: Investigation, data curation; AM-A, EV: Supervision on stress reduction intervention; MT. O-G: Investigation in stress reduction intervention; RC, RE: Supervision on Mediterranean diet intervention; EG: Conceptualization, methodology, writing – original draft, supervision, project administration; M.C: Formal analysis, Supervision; FCr: Conceptualization, methodology, validation, data curation, writing – original draft, supervision, project administration; and all authors: have read and approved the final version of the manuscript.

## Data availability

Individual participant data, study protocol, and statistical analysis plan will be available upon publication. The data will be shared with researchers whose proposed specified use of the data has been approved by the Ethical Committee of the authors’ institute, with a signed data access agreement. To request data, one should contact Francesca Crovetto at francesca.crovetto@sjd.es via email. Raw reads from the gut and vaginal microbiota will be available on NCBI under the accession ID Bioproject.

## Funding

The project was partially funded by a grant from "la Caixa" Foundation (LCF/PR/GN18/10310003); Cerebra Foundation for the Brain Injured Child (Carmarthen, Wales, UK); Departament de Recerca i Universitats de la Generalitat de Catalunya (AGAUR) under grant 2021 SGR Nº 01422; Fundación Mutua Madrileña (AP180722022); and the Instituto de Salud Carlos III (PI22/00684), co-funded by the European Union.

MSR received support from Instituto de Salud Carlos III, through the competitive Sara Borrell fellowship (code CD23/00129) co-funded by the European Union. FC has received support from the Instituto de Salud Carlos III (INT21/00027 and PI20/00246 and PI24/00127) and Fundación para la Investigación Jesus Serra del Grupo Catalana Occidente. SCB has received support from Fundación Alfonso Martín Escudero fellowship. IC has received support from the Instituto de Salud Carlos III (CM23/00118), cofunded by the European Union. ML has received support from Hospital Clínic Barcelona (Contracte Clínic de Recerca Emili Letang-Josep Font). LB has received support from the Instituto de Salud Carlos III (CM21/0058), cofunded by the European Union. LY was supported by the grant FJC2021-048123-I, funded by MCIN/AEI/10.13039/501100011033 and by the European Union “NextGenerationEU”/PRTR”. AN reports personal fees from La Caixa Foundation (Doctoral INPhINIT - RETAINING, fellowship n LCF/BQ/DR19/11740018) and Patrocinio por parte de la Fundacion Mutua Madrile~na en la Financiacion y Desarrollo del proyecto AP180722022 and AP16002/2024.

EG reports that, during the conduct of the study, grants from La Caixa Foundation, grants from Cerebra Foundation for the Brain Injured Child, and grants from AGAUR were received. MCC has received support from Fundación para la Investigación Jesus Serra del Grupo Catalana Occidente and also from the Spanish Ministry of Science and Innovation (MCIN) research grant (ref. PID2022-139475OB-I00). Moreover, MCC acknowledges the award of the Spanish Government MCIN/AEI to the IATA-CSIC as Centre of Excellence Severo Ochoa (CEX2021-001189-S MCIN/AEI/10.13039/ 501100011033). FCr has received support from Instituto de Salud Carlos III (PI22/00684), co-funded by the European Union. The funders had no role in the design and conduct of the study; collection, management, analysis, and interpretation of the data; preparation, review, or approval of the manuscript; and decision to submit the manuscript for publication.

## Conflict of interest

AMA reports grants from La Caixa Foundation during the conduct of the study; as relevant financial activities outside the submitted work, he also reported book royalties from Editorial Planeta, Editorial Plataforma, and from M.ederic Ediciones; conferences from Soc. Andorrana Ciencias and from Fund Instituto Guttmann; finally, he is the founder and owner of Instituto Esmindfulness SL. E.V. reports that, outside the submitted work, received funding from IDIBAPS through the Translational Multidisciplinary Research Program of Disorders of the Brain and personal fees from Abbott, Allergan, Angelini, Lundbeck, Sage, and Sanofi; grants from Dainippon Sumitomo, Ferrer, and Janssen. RE reports grants from the Fundación Dieta Mediterráea (Spain), and Cerveza y Salud (Spain), and personal fees for given lectures from Brewers of Europe (Belgium), the Fundación Cerveza y Salud (Spain), Pernaud-Ricard (Mexico), Instituto Cervantes (Alburquerque, United States), Instituto Cervantes (Milan, Italy), Instituto Cervantes (Tokyo, Japan), Lilly Laboratories (Spain), and the Wine and Culinary International Forum (Spain), as well as nonfinancial support for the organization of a National Congress on Nutrition and feeding trials with products from Grand Fountain and Uriach Laboratories (Spain).

All other authors report no conflicts of interest.

## References

[1] Zmora N., Bashiardes S., Levy M., Elinav E. (2017). The role of the immune system in metabolic health and disease. Cell. Metab.

[2] Nunn K.L., Witkin S.S., Schneider G.M., Boester A., Nasioudis D., Minis E. (2021). Changes in the vaginal microbiome during the pregnancy to postpartum transition. Reprod. Sci..

[3] Rasmussen M.A., Thorsen J., Dominguez-Bello M.G., Blaser M.J., Mortensen M.S., Brejnrod A.D. (2020). Ecological succession in the vaginal microbiota during pregnancy and birth. ISME J..

[4] Gomez-Gallego C., Garcia-Mantrana I., Martinez-Costa C., Salminen S., Isolauri E., Collado M.C. (2019). The microbiota and malnutrition: Impact of nutritional status during early life. Annu. Rev. Nutr..

[5] Calatayud M., Koren O., Collado M.C. (2019). Maternal microbiome and metabolic health program microbiome development and health of the offspring. Trends Endocrinol. Metabol..

[6] Rothschild D., Weissbrod O., Barkan E., Kurilshikov A., Korem T., Zeevi D. (2018). Environment dominates over host genetics in shaping human gut microbiota. Nature.

[7] Hechler C., Borewicz K., Beijers R., Saccenti E., Riksen-Walraven M., Smidt H. (2019). Association between psychosocial stress and fecal microbiota in pregnant women. Sci. Rep..

[8] Selma-Royo M., García-Mantrana I., Calatayud M., Parra-Llorca A., Martínez-Costa C., Collado M.C. (2021). Maternal diet during pregnancy and intestinal markers are associated with early gut microbiota. Eur. J. Nutr..

[9] Crovetto F., Crispi F., Casas R., Martín-Asuero A., Borràs R., Vieta E. (2021). Effects of Mediterranean diet or mindfulness-based stress reduction on prevention of small-for-gestational age birth weights in newborns born to at-risk pregnant individuals: the IMPACT BCN randomized clinical trial. JAMA.

[10] Crovetto F., Crispi F., Borras R., Paules C., Casas R., Martín-Asuero A. (2021). Mediterranean diet, mindfulness-based stress reduction and usual care during pregnancy for reducing fetal growth restriction and adverse perinatal outcomes: IMPACT BCN (Improving Mothers for a better PrenAtal Care Trial BarCeloNa): a study protocol for a randomized controlled trial. Trials.

[11] The investigation and management of the small for gestational age fetus. Royal College of Obstetricians and Gynaecologists. Updated January 2014. Accessed November 11, 2021. https://www.rcog.org.uk/globalassets/documents/guidelines/gtg_31.pdf

[12] Juton C., Castro-Barquero S., Casas R., Freitas T., Ruiz-león A.M., Crovetto F. (2021). Reliability and concurrent and construct validity of a food frequency questionnaire for pregnant women at high risk to develop fetal growth restriction. Nutrients.

[13] Castro-Barquero S., Crovetto F., Estruch R., Ruiz-León A.M., Larroya M., Sacanella E. (2024). Validation of a pregnancy-adapted Mediterranean Diet Adherence Screener (preg-MEDAS): a validation study nested in the Improving Mothers for a better PrenAtal Care Trial BarCeloNa (IMPACT BCN) trial. Am. J. Clin. Nutr..

[14] Klindworth A., Pruesse E., Schweer T., Peplies J., Quast C., Horn M. (2013). Evaluation of general 16S ribosomal RNA gene PCR primers for classical and next-generation sequencing-based diversity studies. Nucleic. Acids Res..

[15] Callahan B.J., McMurdie P.J., Rosen M.J., Han A.W., Johnson A.J.A., Holmes S.P. (2016). DADA2: high-resolution sample inference from Illumina amplicon data. Nat. Methods.

[16] Merra G., Noce A., Marrone G., Cintoni M., Tarsitano M.G., Capacci A. (2021). Influence of Mediterranean diet on human gut microbiota. Nutrients.

[17] Garcia-Mantrana I., Selma-Royo M., Alcantara C., Collado M.C. (2018). Shifts on gut microbiota associated to Mediterranean diet adherence and specific dietary intakes on general adult population. Front. Microbiol..

[18] Kim M.H., Yun K.E., Kim J., Park E., Chang Y., Ryu S. (2020). Gut microbiota and metabolic health among overweight and obese individuals. Sci. Rep..

[19] Gomez-Arango L.F., Barrett H.L., McIntyre H.D., Callaway L.K., Morrison M., Dekker Nitert M. (2016). Increased systolic and diastolic blood pressure is associated with altered gut microbiota composition and butyrate production in early pregnancy. Hypertension.

[20] Jaagura M., Viiard E., Karu-Lavits K., Adamberg K. (2021). Low-carbohydrate high-fat weight reduction diet induces changes in human gut microbiota. Microbiologyopen.

[21] So D., Whelan K., Rossi M., Morrison M., Holtmann G., Kelly J.T. (2018). Dietary fiber intervention on gut microbiota composition in healthy adults: a systematic review and meta-analysis. Am. J. Clin. Nutr..

[22] Miller C.B., Benny P., Riel J., Boushey C., Perez R., Khadka V. (2021). Adherence to Mediterranean diet impacts gastrointestinal microbial diversity throughout pregnancy. BMC Pregnancy Childbirth.

[23] Gur T.L., Shay L., Palkar A.V., Fisher S., Varaljay V.A., Dowd S. (2017). Prenatal stress affects placental cytokines and neurotrophins, commensal microbes, and anxiety-like behavior in adult female offspring. Brain Behav. Immun..

[24] Müller B., Rasmusson A.J., Just D., Jayarathna S., Moazzami A., Novicic Z.K. (2021). Fecal short-chain fatty acid ratios as related to gastrointestinal and depressive symptoms in young adults. Psychosom. Med..

[25] Lynch J.B., Gonzalez E.L., Choy K., Faull K.F., Jewell T., Arellano A. (2023). Gut microbiota Turicibacter strains differentially modify bile acids and host lipids. Nat. Commun..

[26] Jašarević E., Howard C.D., Misic A.M., Beiting D.P., Bale T.L. (2017). Stress during pregnancy alters temporal and spatial dynamics of the maternal and offspring microbiome in a sex-specific manner. Sci. Rep..

[27] Martinez-Aran A., Vieta E. (2022). Precision psychotherapy. Eur. Neuropsychopharmacol..

[28] Houttu N., Mokkala K., Saleem W.T., Virtanen S., Juhila J., Koivuniemi E. (2022). Potential pathobionts in vaginal microbiota are affected by fish oil and/or probiotics intervention in overweight and obese pregnant women. Biomed. Pharmacoth..

[29] Gupta P., Singh M.P., Goyal K. (2020). Diversity of vaginal microbiome in pregnancy: deciphering the obscurity. Front. Public Health.

