# Supplementary material for: Effects of Mediterranean diet or Mindfulness-Based Stress Reduction during pregnancy on maternal gut and vaginal microbiota: a subanalysis of the Improving Mothers for a better PrenAtal Care Trial BarCeloNa (IMPACT BCN) trial
Source: Am J Clin Nutr. 2025 Aug 5;122(4):1121–33. doi: 10.1016/j.ajcnut.2025.07.030 (PMC12674037; doi:10.1016/j.ajcnut.2025.07.030)
Supplement: Supplementary file 1 [file mmc1.pdf]

Effects of Mediterranean diet or Mindfulness Based-Stress Reduction during Pregnancy on Maternal Gut and Vaginal Microbiota. A sub-analysis of the IMPACT BCN trial.

Selma-Royo M. & Crispi F. et al

SUPLEMENTARY DATA

Criteria of high-risk for SGA newborns

Risk factors for small for gestational age were adapted from the Guideline No. 31.2 of the RCOG for our institutional clinical protocol: Major criteria (OR >2) is there was one or more risk factors among the following: maternal age >40ys, Smokers ≥11 cigarettes/day daily, vigorous exercise, drug abuse, previous SGA baby, previous stillbirth, previous preeclampsia <34 weeks, mother born SGA, chronic hypertension, diabetes mellitus, renal diseases, autoimmune diseases, father born SGA, abnormal uterine arteries in the first trimester, high-risk of preeclampsia PAPP-A <0.4 MoM, echogenic bowel, heavy bleeding similar to menses; minor criteria (OR <2) is there was three or more risk factors among the following: : maternal age ≥35ys, Smokers 1-10 cigarettes/day nulliparity, BMI <20, BMI ≥25, previous preeclampsia >34 weeks, pregnancy interval <6months or ≥ 60 months, assisted reproductive technologies

Extended computational methods

Data from the sequencing of both faecal datasets (women assessed only at the final time point and women from the follow-up group; **Figure 1A**) were processed separately with the same pipeline using dada2 algorithm (1) to reduce the potential effect of the different sequencing platform. Basically, quality-trimmed and filtering were assessed after the quality examination. Primers were also removed during this initial trimming

step with the other default parameters. Final forward and reverse reads were merged and chimeric sequenced removed to obtain the final amplicon sequenced variant (ASV) table. Taxonomic assignment was conducted using the Silva v132 database with the addition of the specie level classification for the vaginal dataset (2). Tables at ASV level from both faecal datasets were merged and samples with less than 1000 reads were also removed from the final analysis and were not considered in the final population (n=351).

The effect of the gestational intervention on the maternal microbiota was conducted using RStudio through several packages, mainly phyloseq (3) and vegan (4) packages. Alpha-diversity index both microbial richness and diversity was assessed by the calculation of Observed and Chao1 index and Shannon index, respectively by the phyloseq package. These indexes were calculated on the rarefied data to the minimum sample size for faecal microbiota (reads=23251) and vaginal (reads=34811) datasets. The rarefaction threshold was set considering those samples with number of reads higher than 8000 (n=3 samples were not included for alpha diversity analysis in the faecal dataset).

Differences in beta diversity of the microbial communities were evaluated by the Permutational Multivariate Analysis of Variance based on the Bray-Curtis distance using adonis2 function from the vegan package at Amplicon Sequence Variant (ASV) level. Differences in the overall structure of the microbial community were assessed by Adonis test, controlling by batch as factor, and then visualized using Principal Coordinate analysis (PCoA) plot using Bray-Curtis distance and the Canonical Coordinate analysis (CCA) plots using phyloseq, vegan and ggordiplot package (5). Additionally, principal

components analysis (PCA) on the centered-log ratio transformed (CLR) data at the genus level was also performed to visualize the distribution of the samples according to microbiota composition using FactoMiner and Factoextra package (6,7). For compositional analysis, only those taxa that showed more than 3 reads in at least 20% of the samples were considered. Differential abundance analysis in microbiota composition was assessed through general linear models by Maaslin2 R package (8) adjusted by pre-pregnancy body mass index (BMI, kg/m^2) and antibiotics use during pregnancy (Yes/No) as covariates. All multiple comparisons were adjusted using the Benjamini Hochberg method and FDR adjusted p-value is expressed as q-value in the manuscript (q-value <0.2 is considered for statistical significance). Spearman's rank correlation was performed to find potential relation between specific foods and taxa from the maternal gut microbiome. For beta diversity and compositional analysis, ConQuR package was used to remove the potential bias associated with the sequencing batch (9). Furthermore, the batch variable was also included as a factor in the general lineal models.

VALENCIA (VAginaL community state typE Nearest CentroId clAssifier) tool was used to assess the classification of the mothers based on their community state type (CST) from their vaginal microbiota composition (10). All graphs were plotted using the following packages: ggplot2 (11), ggpubr (12), forcats (13).

Table S1. Maternal consumption of key dietary food intake at baseline and final according to intervention groups.

		Within-group mean changes				Between-group changes		
		Usual care n=105	Mediterranean diet n=98	Stress reduction n=111	P^3	MedDiet *vs.* Usual care *Difference (95% CI)*	Stress reduction *vs.* Usual care *Difference (95% CI)*	MedDiet *vs.* Stress reduction *Difference (95% CI)*
Extra-virgin Olive Oil - g/d	Baseline[1]	30.1 ± 19.5	33.7 ± 18.2	33.1 ± 19.0				
	Final[2]	35.0 ± 1.55*	45.6 ± 1.60**	36.3 ± 1.50*	<0.001	10.6 (6.24 to 15.0)	1.35 (-2.88 to 5.60)	9.26 (4.96 to 13.6)
Refined olive oil - g/d	Baseline	8.36 ± 16.1	8.06 ± 15.1	8.36 ± 13.6				
	Final	5.90 ± 1.18	1.61 ± 1.22**	8.44 ± 1.15	<0.001	-4.29 (-7.61 to -0.98)	2.54 (-0.68 to 5.76)	-6.83 (-10.1 to -3.55)
Total nuts - g/d	Baseline	18.8 ± 18.6	21.1 ± 19.4	21.7 ± 22.4				
	Final	18.6 ± 1.68	33.6 ± 1.76**	18.1 ± 1.64	<0.001	14.9 (10.2 to 19.7)	-0.49 (-5.10 to 4.11)	15.4 (10.7 to 20.1)
Vegetables - g/d	Baseline	295.0 ± 135.9	285.0 ± 129.1	301.6 ± 129.0				
	Final	285.3 ± 9.58	341.4 ± 9.98**	276.7 ± 9.32*	<0.001	64.6 (37.9 to 91.4)	-8.55 (-34.8 to 17.7)	64.6 (37.9 to 91.4)
Legumes (g/d)	Baseline	49.1 ± 36.5	56.7 ± 39.6	48.9 ± 39.7				
	Final	51.1 ± 3.67	82.4 ± 3.81**	49.6 ± 3.59	<0.001	31.3 (20.9 to 41.6)	-1.48 (-11.5 to 8.58)	32.7 (22.5 to 43.0)
Fruits (g/d)	Baseline	331.6 ± 195.6	328.4 ± 152.9	341.4 ± 174.6				
	Final	334.4 ± 19.0*	419.9 ± 19.5**	337.1 ± 18.2	0.002	85.5 (32.2 to 138.8)	2.70 (-48.8 to 54.2)	82.8 (30.6 to 135.0)
Refined cereals (g/d)	Baseline	71.8 ± 45.2	61.4 ± 40.8	62.8 ± 50.0				
	Final	59.6 ± 3.59*	31.3 ± 3.68**	57.9 ± 3.46	<0.001	-28.4 (-38.5 to -18.3)	-1.76 (-11.6 to 8.03)	-26.6 (-36.5 to -16.7)
Whole grain cereals (g/d)	Baseline	39.6 ± 43.4	38.2 ± 40.7	40.7 ± 44.1				
	Final	38.4 ± 3.12	64.4 ± 3.24**	42.7 ± 3.08	<0.001	25.9 (17.1 to 34.7)	4.21 (-4.40 to 12.8)	21.7 (12.9 to 30.5)
Fish or seafood (g/d)	Baseline	71.0 ± 39.4	73.6 ± 41.6	74.4 ± 40.8				
	Final	74.3 ± 3.79	104.2 ± 3.95**	81.1 ± 3.74	<0.001	29.9 (19.1 to 40.6)	6.87 (-3.56 to 17.3)	23.0 (12.4 to 33.7)
Fat fish (g/d)	Baseline	14.1 ± 16.8	14.0 ± 15.5	16.4 ± 17.4				
	Final	15.6 ± 1.74	32.6 ± 1.79**	19.9 ± 1.69*	<0.001	17.0 (12.1 to 21.9)	4.27 (-0.48 to 9.02)	12.7 (7.90 to 17.6)
Lean meat (g/d)	Baseline	66.3 ± 37.3	76.4 ± 37.5	65.2 ± 34.5				
	Final	66.1 ± 3.08	79.7 ± 3.21*	69.6 ± 3.01	0.007	13.6 (4.82 to 22.3)	3.47 (-4.96 to 11.9)	10.1 (1.43 to 18.7)
Red meat (g/d)	Baseline	49.6 ± 37.8	51.0 ± 36.9	48.9 ± 32.3				
	Final	48.9 ± 2.76	38.4 ± 2.87*	49.9 ± 2.70	0.006	-10.6 (-18.4 to -2.77)	0.98 (-6.59 to 8.55)	-11.5 (-19.3 to -3.82)
Processed meat (g/d)	Baseline	34.7 ± 25.1	30.8 ± 21.3	40.5 ± 44.0				
	Final	29.2 ± 2.01*	36.2 ± 2.09	30.6 ± 1.96*	0.039	7.01 (1.32 to 12.7)	1.41 (-4.09 to 6.91)	5.60 (-0.03 to 11.2)
Pastries. cakes or sweets (g/d)	Baseline	46.4 ± 34.1	38.7 ± 39.3	39.4 ± 47.2				
	Final	38.8 ± 2.82	28.4 ± 2.89*	40.8 ± 2.72	0.004	-10.4 (-18.3 to -2.44)	2.01 (-5.71 to 9.72)	-12.4 (-20.2 to -4.59)
Dairy products (g/d)	Baseline	370.6 ± 263.7	327.6 ± 231.7	367.4 ± 225.2				
	Final	343.4 ± 17.4	471.3 ± 17.8**	361.0 ± 17.1	<0.001	127.9 (79.1 to 176.6)	17.5 (-30.3 to 65.4)	110.3 (61.7 to 158.9)
MedDiet Score	Baseline	7.29 ± 2.55	8.12 ± 2.49	7.96 ± 2.78				
	Final	7.90 ± 0.21	12.3 ± 0.22**	8.38 ± 0.20*	<0.001	4.37 (3.78 to 4.97)	0.48 (-0.09 to 1.05)	3.89 (3.30 to 4.48)

MedDiet: Mediterranean diet; EVOO: Extra Virgin Olive Oil.
[1]Baseline values are observed means ± SD. [2] Final values are baseline-adjusted (least-squares) means ± SE and comparison among groups done with ANCOVA analysis. *P<0.05 and **P<0.001 final from baseline comparison. [3] ANCOVA analysis.
Data available for 314 pregnancies.

Table S2. Maternal dietary energy and nutrient intake at baseline and final according to intervention groups.

		Within-group mean changes				Between-group changes		
		Usual care n=98	Mediterranean diet n=105	Stress reduction n=111	*P*[3]	MedDiet *vs.* Usual care *Difference*	Stress reduction *vs.* Usual care *Difference*	MedDiet *vs.* Stress reduction *Difference*
Energy - kcal/d	Baseline[1]	2577 ± 538.0	2481 ± 533.9	2487.± 528.2				
	Final[2]	2402 ± 42.4*	2656 ± 43.8*	2460 ± 41.2	<0.001	254.0 (134.5 to 373.5)	57.6 (-58.3 to 173.6)	196.3 (78.5 to 314.2)
Protein - g/d	Baseline	106.6 ± 25.0	106.2 ± 24.9	107.0 ± 25.2				
	Final	101.5 ± 2.12	121.9 ± 2.19**	105.4 ± 2.06	<0.001	20.5 (14.5 to 26.5)	3.98 (-1.81 to 9.77)	16.5 (10.6 to 22.4)
Carbohydrate - g/d	Baseline	241.9 ± 68.9	219.1 ± 62.2	218.6 ± 66.0				
	Final	213.8 ± 4.71*	220.2 ± 4.84	213.9 ± 4.56	0.556	6.38 (-6.91 to 19.7)	0.10 (-12.8 to 13.0)	6.28 (-6.73 to 19.3)
Fiber - g/d	Baseline	34.4 ± 11.5	33.7 ± 9.68	33.2 ± 11.1				
	Final	32.1 ± 0.86	39.0 ± 0.89**	32.1 ± 0.84	<0.001	6.88 (4.44 to 9.31)	-0.03 (-2.39 to 2.33)	6.90 (4.50 to 9.31)
Total fat - g/d	Baseline	131.3 ± 29.6	130.8 ± 31.7	131.4 ± 31.8				
	Final	126.8 ± 2.62	142.8 ± 2.71**	131.1 ± 2.55	<0.001	16.0 (8.64 to 23.4)	4.28 (-2.88 to 11.4)	11.7 (4.46 to 19.0)
SFA - g/d	Baseline	36.4 ± 10.5	35.3 ± 12.0	36.5 ± 10.7				
	Final	34.5 ± 0.83	37.1 ± 0.85	36.0 ± 0.80	0.097	2.55 (0.22 to 4.88)	1.47 (-0.79 to 3.73)	1.09 (-1.21 to 3.39)
MUFA - g/d	Baseline	61.8 ± 15.4	62.7 ± 15.1	63.1 ± 15.7				
	Final	60.6 ± 1.31	67.2 ± 1.36*	64.3 ± 1.27	0.002	6.60 (2.90 to 10.3)	3.61 (0.02 to 7.20)	2.99 (-0.65 to 6.64)
PUFA - g/d	Baseline	23.4 ± 8.11	23.0 ± 7.58	22.1 ± 7.62				
	Final	22.2 ± 0.70	27.9 ± 0.72**	21.1 ± 0.68	<0.001	5.68 (3.72 to 7.65)	-1.11 (-3.02 to 0.79)	6.80 (4.86 to 8.73)
α-Linoleic acid - g/d	Baseline	15.7 ± 6.42	14.7 ± 5.42	14.2 ± 5.32				
	Final	14.7 ± 0.53*	18.2 ± 0.55**	14.0 ± 0.52	<0.001	3.42 (1.92 to 4.92)	-0.72 (-2.18 to 0.73)	4.15 (2.67 to 5.62)
α-Linolenic acid - g/d	Baseline	1.44 ± 0.62	1.50 ± 0.59	1.44 ± 0.68				
	Final	1.31 ± 0.05*	2.29 ± 0.06**	1.34 ± 0.05*	<0.001	0.98 (0.82 to 1.13)	0.03 (-0.12 to 0.18)	0.95 (0.80 to 1.10)
EPA - g/d	Baseline	0.15 ± 0.11	0.16 ± 0.10	0.16 ± 0.11				

	Final	0.16 ± 0.01	0.27 ± 0.01**	0.18 ± 0.01*	<0.001	0.10 (0.07 to 0.13)	0.02 (-0.01 to 0.05)	0.08 (0.05 to 0.11)
DHA - g/d	Baseline	0.31 ± 0.25	0.31 ± 0.25	0.34 ± 0.27				
	Final	0.33 ± 0.02	0.59 ± 0.03**	0.39 ± 0.02	<0.001	0.25 (0.18 to 0.33)	0.06 (-0.01 to 0.13)	0.19 (0.13 to 0.26)
Trans-FA - g/d	Baseline	1.73 ± 1.09	1.91 ± 1.41	1.57 ± 1.03				
	Final	1.71 ± 0.08	1.31 ± 0.09**	1.56 ± 0.08	0.005	-0.39 (-0.63 to -0.15)	-0.15 (-0.38 to 0.08)	-0.25 (-0.48 to -0.01)
Cholesterol - mg/d	Baseline	345.9 ± 104.1	344.9 ± 114.5	340.2 ± 99.8				
	Final	329.1 ± 8.88	360.2 ± 9.19*	341.9 ± 8.64	0.051	31.0 (6.01 to 56.1)	12.8 (-11.5 to 37.1)	18.3 (-6.46 to 43.0)

MedDiet denotes for Mediterranean diet. MBSR Mindfulness-Based Stress Reduction. SFA Saturated fatty acids. MUFA Monounsaturated fatty acids. PUFA Polyunsaturated fatty acids. EPA Eicosapentaenoic acid. DHA Docosahexaenoic acid and FA Fatty acids.

[1]Baseline values are observed means ± SD. [2] Final values are baseline-adjusted (least-squares) means ± SE and comparison among groups done with ANCOVA analysis. *P<0.05 and **P<0.001 final from baseline comparison. [3] ANCOVA analysis.

Data available for 314 pregnancies.

Table S3. Maternal anxiety and wellbeing evaluated by validated questionnaires at baseline and final according to intervention groups.

		Within-groups mean changes				Between-groups changes		
		Usual care<br>n=120	Mediterranean diet<br>n=109	Stress reduction<br>n=122	*P*[3]	MedDiet *vs.* Usual care<br>*Difference*<br>*(95% CI)*	Stress reduction *vs.* Usual care<br>*Difference*<br>*(95% CI)*	Stress reduction *vs.* MedDiet<br>*Difference*<br>*(95% CI)*
Perceived stress scale score	Baseline	16 ± 7.3	16.7 ± 8.0	16.1± 7.5				
	Final	16.6 ± 0.5	15.6 ± 0.5	16.7 ± 0.5	0.267	-1.02 (-2.46 to 0.43)	0.06 (-1.33 to 1.44)	1.07 (-0.36 to 2.5)
State-trait Anxiety Inventory (anxiety)	Baseline	14.4 ± 9.2	13.9 ± 9	14.1 ± 8.4				
	Final	15.9 ± 0.6*	14 ± 0.6	12.7 ± 0.6*	<0.001	-1.87 (-3.58 to -0.16)	-3.17 (-4.81 to -1.53)	-1.30 (-3 to 0.4)
State-trait Anxiety Inventory (personality)	Baseline	16.1 ± 8.9	14.32± 8.2	15.4 ± 8.3				
	Final	15.4 ± 0.5	15.1 ± 0.6	14.2 ± 0.5	0.217	-0.26 (-1.77 to 1.24)	-1.22 (-2.65 to 0.22)	-0.96 (-2.44 to 0.53)
Five well-being index	Baseline	62.8 ± 16.9	68.6 ± 14.2*	65.5 ± 16.3				
	Final	65 ± 1.2	63.9 ± 1.3	67.5 ± 1.2	0.118	-1.13 (-4.72 to 2.45)	2.46 (-0.95 to 5.87)	3.59 (0.06 to 7.12)
FFMQ: Observation	Baseline	23 ± 6	24.5 ± 6.2	23 ± 6				
	Final	24.4 ± 0.4*	24.2 ± 0.5	28.3 ± 0.4**	<0.001	-0.13 (-1.40 to 1.13)	3.92 (2.72 to 5.12)	4.05 (2.80 to 5.31)
FFMQ 2: Description	Baseline	32 ± 5.7	32.5 ± 5.3	31.5 ± 5.7				
	Final	32.1 ± 0.4	32.2 ± 0.4	32.6 ± 0.4*	0.532	0.17 (-0.91 to 1.25)	0.58 (-0.45 to 1.61)	0.41 (-0.67 to 1.48)
FFMQ 3: Awareness	Baseline	31 .2 ± 6.3	31.1 ± 6.5	30 ± 6.6				
	Final	30.2 ± 0.5	30.4 ± 0.6	30.4 ± 0.5	0.965	0.16 (-1.34 to 1.66)	0.18 (-1.26 to 1.62)	0.02 (-1.48 to 1.52)
FFMQ 4: Non-judgmental	Baseline	29.8 ± 6	29.9 ± 5.3	29.3 ± 5.9				
	Final	29.3 ± 0.4	29.7 ± 0.5	31.1 ± 0.4*	0.008	0.40 (-0.80 to 1.61)	1.75 (0.61 to 2.90)	1.35 (0.15 to 2.55)
FFMQ 5: Non-reactivity	Baseline	22.45± 4.6	23 ± 5.5	21.7 ± 4.8				
	Final	22.9 ± 0.5	22.8 ± 0.6	24.2 ± 0.4**	<0.001	-0.39 (-1.45 to 0.67)	1.77 (0.75 to 2.78)	2.16 (1.09 to 3.22)

MedDiet: Mediterranean diet; FFMQ Five Facet Mindfulness Questionnaire.
[1]Baseline values are observed means ± SD. [2] Final values are baseline-adjusted (least-squares) means ± SE and comparison among groups done with ANCOVA analysis.
*P<0.05 and **P<0.001 final from baseline comparison. [3] ANCOVA analysis.

Table S4. Multivariable model for the gut microbiota composition at phylum and genus level from women classified as high adherence to Mediterranean diet (N=98) based on food frequency questionary compared to those with low adherence (N=53).

	Coeff	95CI	SE	Prev	pval	qval
Phylum						
Epsilonbacteraeota	-0.59	(-1.18; -0.01)	0.30	98.68	0.05	0.196
Synergistetes	1.2	(0.32; 2.09)	0.45	51.66	0.009	0.059
Tenericutes	1.75	(0.9; 2.6)	0.43	57.62	<0.001	0.001
Genus						
Akkermansia	1.24	(-0.06; 2.54)	0.66	71.52	0.063	0.194
Alistipes	1.07	(0.23; 1.9)	0.43	98.01	0.013	0.070
Anaerostipes	1.38	(0.63; 2.13)	0.38	96.69	<0.001	0.006
Bacteroides	0.82	(0.19; 1.45)	0.32	100	0.012	0.066
Blautia	1.11	(0.45; 1.76)	0.33	98.68	0.001	0.012
Butyricicoccus	0.99	(0.05; 1.93)	0.48	88.74	0.042	0.153
Butyricimonas	0.48	(-0.01; 0.96)	0.25	43.71	0.056	0.183
CAG-873	-0.33	(-0.59; -0.07)	0.13	22.52	0.014	0.073
Campylobacter	-0.59	(-1.17; 0)	0.3	98.68	0.051	0.177
Christensenellaceae_R-7_group	1	(0.15; 1.84)	0.43	94.04	0.023	0.101
Clostridium_sensu_stricto_1	1.15	(0.08; 2.22)	0.55	84.77	0.036	0.141
Coprobacillus	0.42	(-0.03; 1.85)	0.48	85.43	0.059	0.187
Collinsella	0.91	(-0.02; 0.86)	0.22	18.54	0.063	0.194
Coprobacter	1.25	(0.46; 2.04)	0.4	47.02	0.002	0.020
Coprococcus_2	1.15	(-0.01; 2.32)	0.59	58.94	0.054	0.183
Coprococcus_3	1.04	(0.01; 2.07)	0.53	89.4	0.05	0.174
Dorea	0.9	(0.2; 1.59)	0.36	98.68	0.013	0.068
Eggerthella	0.69	(0.13; 1.26)	0.29	32.45	0.017	0.086
Erysipelotrichaceae_UCG-003	1.24	(0.19; 2.29)	0.54	92.05	0.022	0.101
Faecalibacterium	0.92	(0.17; 1.67)	0.38	98.01	0.018	0.086
Family_XIII_AD3011_group	0.84	(0.05; 1.63)	0.4	80.13	0.038	0.143
Family_XIII_UCG-001	1.11	(0.32; 1.91)	0.41	87.42	0.007	0.047
Intestinibacter	1.38	(0.36; 2.4)	0.52	85.43	0.009	0.053
Intestinimonas	1.05	(-0.03; 2.15)	0.56	78.81	0.059	0.189
Jonquetella	1.38	(0.51; 2.25)	0.44	45.03	0.002	0.020
Lachnoclostridium	1.2	(0.43; 1.96)	0.39	92.05	0.003	0.022
Lachnospira	1.31	(0.4; 2.21)	0.46	96.03	0.005	0.039
Lachnospiraceae_FCS020_group	1.29	(0.52; 2.06)	0.39	78.81	0.001	0.013
Lachnospiraceae_ND3007_group	1.74	(0.75; 2.74)	0.51	94.04	<0.001	0.009
Lachnospiraceae_NK4A136_group	1.75	(0.76; 2.74)	0.51	94.04	<0.001	0.009
Lachnospiraceae_UCG-001	1.39	(0.4; 2.39)	0.51	69.54	0.007	0.047
Lachnospiraceae_UCG-004	1.40	(0.59; 2.19)	0.41	90.73	<0.001	0.009
Lachnospiraceae_UCG-008	1.52	(0.83; 2.22)	0.36	44.37	<0.001	<0.001
Lactococcus	1.29	(0.52; 2.07)	0.4	59.6	0.001	0.013
Marvinbryantia	1.04	(0.16; 1.93)	0.45	86.09	0.023	0.101
Megamonas	-0.94	(-1.34; -0.54)	0.2	25.83	<0.001	<0.001
Merdibacter	1.18	(0.3; 2.06)	0.45	62.25	0.009	0.056
Odoribacter	0.99	(0.15; 1.82)	0.43	83.44	0.022	0.101

Parabacteroides	0.91	(0.12; 1.71)	0.41	96.69	0.026	0.110
Peptostreptococcus	-1.43	(-2.76; -0.1)	0.68	92.05	0.037	0.142
Prevotellaceae_Ga6A1_group	-0.67	(-1.12; -0.22)	0.23	49.01	0.004	0.03
Pyramidobacter	0.78	(-0.01; 1.57)	0.4	41.72	0.055	0.183
Roseburia	1.61	(0.56; 2.65)	0.53	87.42	0.003	0.026
Ruminiclostridium_6	1.49	(0.33; 2.64)	0.59	82.12	0.012	0.068
Ruminiclostridium_9	0.83	(-0.01; 1.67)	0.43	86.09	0.055	0.183
Ruminococcaceae_NK4A214_group	0.96	(0.26; 1.66)	0.36	94.04	0.008	0.050
Ruminococcaceae_UCG-010	1.24	(0.42; 2.04)	0.41	59.6	0.003	0.026
Senegalimassilia	1.61	(0.78; 2.45)	0.42	80.13	<0.001	0.004
Solobacterium	-1.53	(-2.57; -0.49)	0.53	47.02	0.005	0.034
Subdoligranulum	1.30	(0.41; 2.19)	0.45	96.69	0.005	0.036
W5053	1.03	(0.31; 1.75)	0.37	29.8	0.006	0.040

Multivariable analysis was performed using the Maaslin2 algorithm with the total sum scaling (TSS) normalization and log transformation. Pre-pregnancy body mass index and antibiotics were also included in the model as potential confounding factors. Those samples classified as "Medium" for the Mediterranean diet category were removed from this analysis. Only those genera with an FDR (q-value) <0.2 are shown in the table.

Table S5. Multivariable model for the gut microbiota composition at phylum and genus level from women classified as High stress based on 5 well-being questionaries (WHO-5 index <55, N=60) compared to those with low stress (WHO-5 index >75, N=109).

	Coeff	95CI	SE	Prev	pval	q-val
Phylum						
Epsilonbacteraeota	0.67	(0,16; 1,19)	0.26	99.41	0.011	0.067
Genus						
Arcanobacterium	0.89	(0.06; 1.74)	0.43	42.01	0.037	0.15
Bacteroides	-0.71	(-1.29; -0.14)	0.29	100	0.016	0.091
Bilophila	-1.37	(-2.3; -0.42)	0.48	78.7	0.005	0.04
Blautia	-0.63	(-1.23; -0.02)	0.31	98.82	0.045	0.169
Brevibacterium	-1.27	(-2.23; -0.3)	0.49	46.75	0.011	0.069
Campylobacter	0.67	(0.16; 1.18)	0.26	99.41	0.011	0.069
Faecalibacterium	-0.81	(-1.54; -0.06)	0.38	98.82	0.036	0.144
Family_XIII_UCG-001	-0.85	(-1.56; -0.14)	0.36	90.53	0.021	0.106
Lachnoclostridium	-0.73	(-1.47; 0.02)	0.38	92.9	0.057	0.197
Parabacteroides	-0.73	(-1.44; -0.02)	0.36	96.45	0.046	0.173
Ruminococcaceae_UCG-004	-1.35	(-2.5; -0.18)	0.59	73.96	0.025	0.12
Ruminococcaceae_UCG-010	-0.79	(-1.58; 0)	0.4	63.31	0.051	0.184
Sellimonas	-0.59	(-1.18; 0.01)	0.31	47.34	0.057	0.197

Multivariable analysis was performed using the Maaslin2 algorithm with the total sum scaling (TSS) normalization and log transformation. Pre-pregnancy body mass index and antibiotics were also included in the model as potential confounding factors. Those samples classified as "Medium" for the Stress index category were removed from this analysis (5-well-being index between 55-75). Only those genera with an FDR (q-value) <0.2 are shown in the table.

SUPPLEMENTARY FIGURES

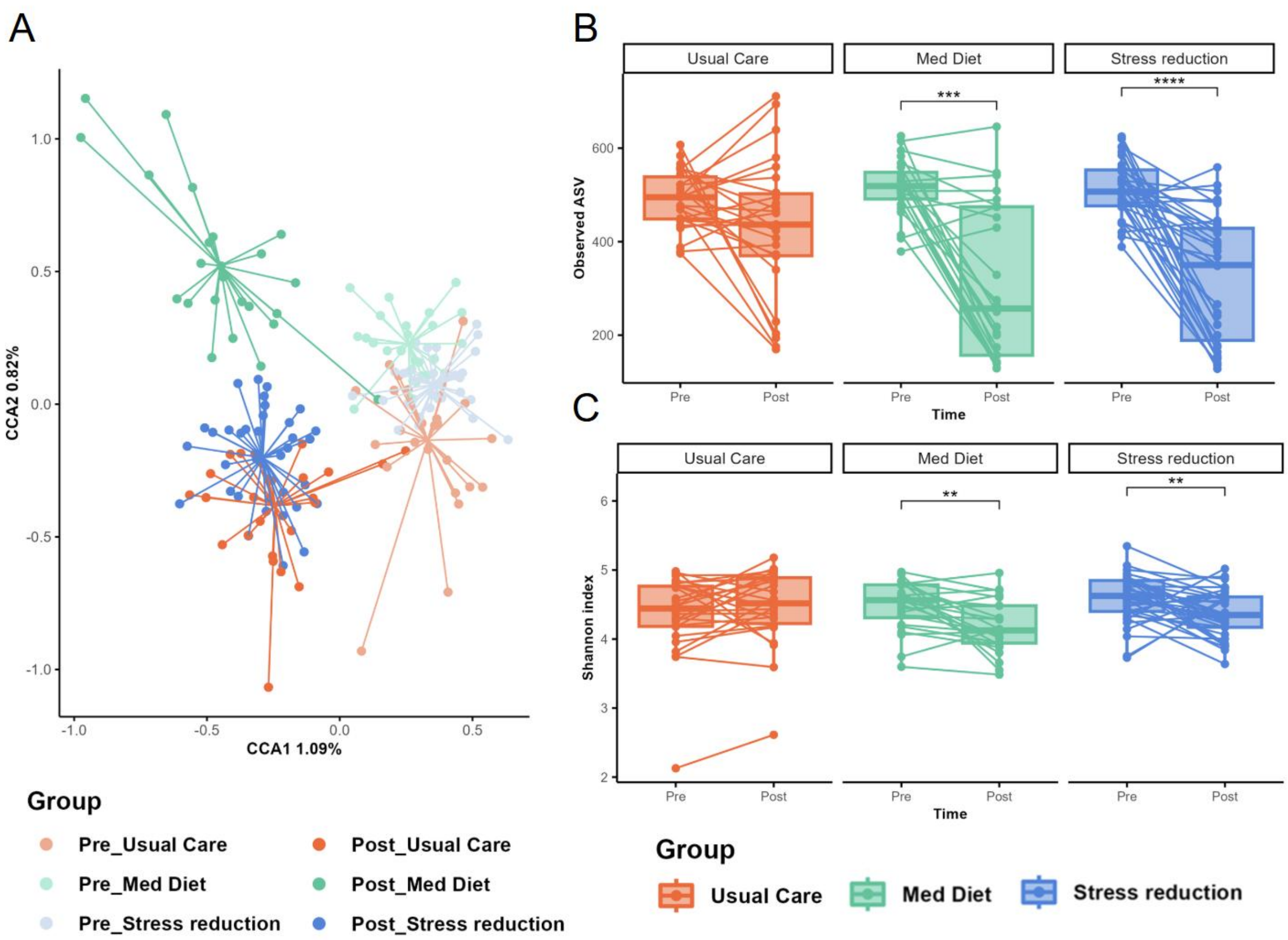

Figure S1. Effect of the interventions on the composition and diversity of gut microbiota from pregnant women at baseline and the end of intervention (n=85). A) Canonical coordinate analysis of the pregnant women according to gestational intervention and time of sample collection (Pre intervention vs Post intervention) (CCA F=1.1170, p=0.001). **B-C**) Effect of the time of collection (Pre and Post) and gestational interventions on the alpha diversity of the gut microbiota measured as Observed amplicon sequence variance (ASV) (B) and Shannon index (C). Comparisons between the groups were assessed by the Kruskal-Wallis test after distribution testing.

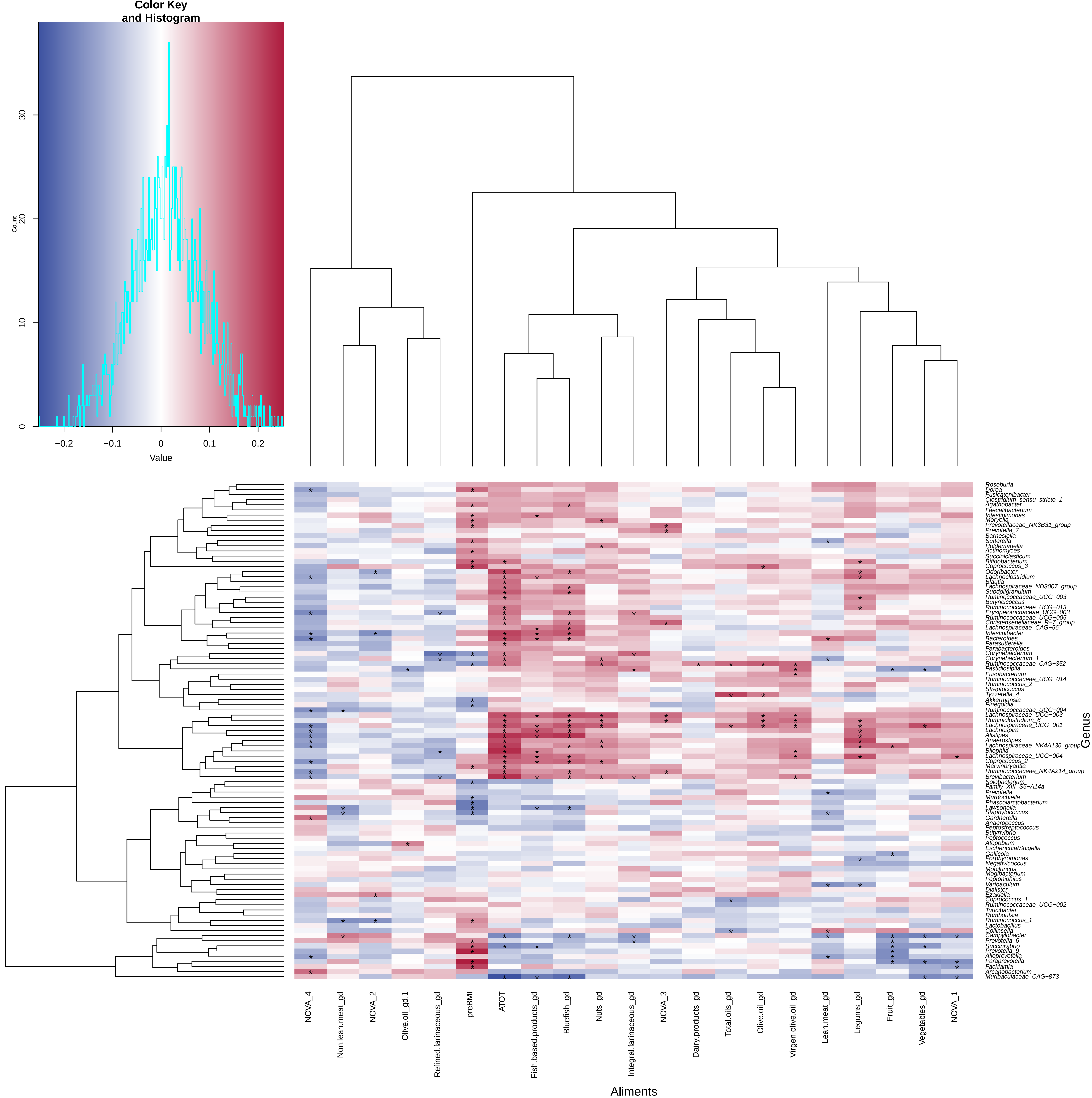

Color Key
and Histogram
Count
Value
−0.2
−0.1
0
0.1
0.2
0
10
20
30
Roseburia
Dorea
Fusicatenibacter
Clostridium_sensu_stricto_1
Agathobacter
Faecalibacterium
Intestinimonas
Moryella
Prevotellaceae_NK3B31_group
Prevotella_7
Barnesiella
Sutterella
Holdemanella
Actinomyces
Succiniclasticum
Bifidobacterium
Coprococcus_3
Odoribacter
Lachnoclostridium
Blautia
Lachnospiraceae_ND3007_group
Subdoligranulum
Ruminococcaceae_UCG−003
Butyricicoccus
Ruminococcaceae_UCG−013
Erysipelotrichaceae_UCG−003
Ruminococcaceae_UCG−005
Christensenellaceae_R−7_group
Lachnospiraceae_CAG−56
Intestinibacter
Bacteroides
Parasutterella
Parabacteroides
Corynebacterium
Corynebacterium_1
Ruminococcaceae_CAG−352
Fastidiosipila
Fusobacterium
Ruminococcaceae_UCG−014
Ruminococcus_2
Streptococcus
Tyzzerella_4
Akkermansia
Finegoldia
Ruminococcaceae_UCG−004
Lachnospiraceae_UCG−003
Ruminiclostridium_6
Lachnospiraceae_UCG−001
Lachnospira
Alistipes
Anaerostipes
Lachnospiraceae_NK4A136_group
Bilophila
Lachnospiraceae_UCG−004
Coprococcus_2
Marvinbryantia
Ruminococcaceae_NK4A214_group
Brevibacterium
Solobacterium
Family_XIII_S5−A14a
Prevotella
Murdochiella
Phascolarctobacterium
Lawsonella
Staphylococcus
Gardnerella
Anaerococcus
Peptostreptococcus
Butyrivibrio
Peptococcus
Atopobium
Escherichia/Shigella
Gallicola
Porphyromonas
Negativicoccus
Mobiluncus
Mogibacterium
Peptoniphilus
Varibaculum
Dialister
Ezakiella
Coprococcus_1
Ruminococcaceae_UCG−002
Turicibacter
Romboutsia
Ruminococcus_1
Lactobacillus
Collinsella
Campylobacter
Prevotella_6
Succinivibrio
Prevotella_9
Alloprevotella
Paraprevotella
Facklamia
Arcanobacterium
Muribaculaceae_CAG−873
Genus
NOVA_4
Non.lean.meat_gd
NOVA_2
Olive.oil_gd.1
Refined.farinaceous_gd
preBMI
ATOT
Fish.based.products_gd
Bluefish_gd
Nuts_gd
Integral.farinaceous_gd
NOVA_3
Dairy.products_gd
Total.oils_gd
Olive.oil_gd
Virgen.olive.oil_gd
Lean.meat_gd
Legums_gd
Fruit_gd
Vegetables_gd
NOVA_1
Aliments

Figure S2. Heatmap of the Spearman's correlation between analyzed dietary foods and selected taxa from pregnant women's gut microbiota at the genus level. Those taxa with an average relative abundance higher than 5% across all samples were included in the correlation analysis. Food intake is expressed as g/day (gd) and bacteria as relative abundance (%).

REFERENCES

1. Callahan BJ, McMurdie PJ, Rosen MJ, Han AW, Johnson AJ, Holmes SP. DADA2: High-resolution sample inference from Illumina amplicon data. Nat Methods. 2016 Jul;13(7):581-3. doi: 10.1038/nmeth.3869

2. Quast C, Pruesse E, Yilmaz P, Gerken J, Schweer T, Yarza P, Peplies J, Glöckner FO (2013) The SILVA ribosomal RNA gene database project: improved data processing and web-based tools. Nucl. Acids Res. 41 (D1): D590-D596.

3. McMurdie PJ, Holmes S. phyloseq: An R Package for Reproducible Interactive Analysis and Graphics of Microbiome Census Data. Watson M, ed. *PLoS ONE*. 2013;8(4):e61217. doi:10.1371/journal.pone.0061217

4. Dixon P. VEGAN, a package of R functions for community ecology. *J Veg Sci*. 2003;14(6):927-930. doi:10.1111/j.1654-1103.2003.tb02228.x

5. John Quensen. Make ggplot Versions of Vegan's Ordiplots. R package version 0.4.1. Published online 2021. http://github.com/jfq3/ggordiplots

6. Alboukadel Kassambara and Fabian Mundt. factoextra: Extract and Visualize the Results of Multivariate Data Analyses. R package version 1.0.7. Published online 2020. https://CRAN.R-project.org/package=factoextra

7. Sebastien Le, Julie Josse, Francois Husson. FactoMineR: An R Package for Multivariate Analysis. Journal of Statistical Software, 25(1), 1-18. 10.18637/jss.v025.i01.

8. Dhariwal A, Chong J, Habib S, King IL, Agellon LB, Xia J. MicrobiomeAnalyst: a web-based tool for comprehensive statistical, visual and meta-analysis of microbiome data. *Nucleic Acids Res*. 2017;45(W1):W180-W188. doi:10.1093/nar/gkx295

9. Ling W, Lu J, Zhao N, et al. Batch effects removal for microbiome data via conditional quantile regression. *Nat Commun*. 2022;13(1):5418. doi:10.1038/s41467-022-33071-9

10. France MT, Ma B, Gajer P, et al. VALENCIA: a nearest centroid classification method for vaginal microbial communities based on composition. *Microbiome*. 2020;8(1):166. doi:10.1186/s40168-020-00934-6

11. Ginestet C. ggplot2: Elegant Graphics for Data Analysis: Book Reviews. *J R Stat Soc Ser A Stat Soc*. 2011;174(1):245-246. doi:10.1111/j.1467-985X.2010.00676_9.x

12. Alboukadel Kassambara. ggpubr: "ggplot2" Based Publication Ready Plots. R package version 0.4.0. Published online 2020. https://CRAN.R-project.org/package=ggpubr

13. Hadley Wickham. Tools for Working with Categorical Variables (Factors). R package version 0.5.1. Published online 2021. https://CRAN.R-project.org/package=forcats
